# A poly-δ-decalactone (PDL) based nanoemulgel for topical delivery of ketoconazole and eugenol against *Candida albicans*†

**DOI:** 10.1039/d4na00176a

**Published:** 2024-08-02

**Authors:** Prashant Dubey, Ankaj Kumar, Klaudi K. Vaiphei, Sargun Basrani, Ashwini Jadhav, Carl-Eric Wilen, Jessica M. Rosenholm, Kuldeep K. Bansal, Rudra Chakravarti, Dipanjan Ghosh, Arvind Gulbake

**Affiliations:** a Department of Pharmaceutics, National Institute of Pharmaceutical Education and Research Guwahati Assam 781101 India arvind@niperguwahati.in arvind.gulbake@gmail.com; b Department of Medical Biotechnology, CIR, D.Y. Patil Education Society, Institution Deemed to be University Kolhapur India; c Laboratory of Molecular Science and Engineering, Åbo Akademi University Aurum, Henrikinkatu 2 20500 Turku Finland kuldeep.bansal@abo.fi; d Pharmaceutical Sciences Laboratory, Faculty of Science and Engineering, Åbo Akademi University Turku 20520 Finland; e Department of Natural Products, National Institute of Pharmaceutical Education and Research Kolkata India

## Abstract

This study aimed to investigate the potential of poly-δ-decalactone (PDL) and a block copolymer (methoxy-poly(ethylene glycol)-*b*-poly-δ-decalactone (mPEG-*b*-PDL)) in the topical delivery of ketoconazole (KTZ) and eugenol (EUG) against *Candida albicans*. The nanoemulsion (NE) was studied for its significant factors and was optimized using the design of experiments (DOE) methodologies. A simple robust nanoprecipitation method was employed to successfully produce a nanoemulsion (KTZ–EUG–NE). The spherical globules exhibited rough surfaces, explaining the adsorption of mPEG-*b*-PDL onto PDL. The sustained drug release effects were governed by the amorphous nature of PDL. KTZ–EUG–NE was further used to develop a 1% w/v Carbopol-940-based nanoemulgel (KTZ–EUG–NE gel). The optimal rheological and spreadability properties of the developed nanoemulgel explain the ease of topical applications. *Ex vivo* permeation and retention studies confirmed the accumulation of KTZ–EUG–NE at different layers of the skin when applied topically. The cytotoxicity of the developed NE in human keratinocyte (HaCaT) cells demonstrated the utility of this newly explored nanocarrier in reducing the cell toxicity of KTZ. The higher antifungal activities of KTZ–EUG–NE at 19.23-fold lower concentrations for planktonic growth and 4-fold lower concentrations for biofilm formation than coarse drugs explain the effectiveness of the developed NE.

## Abstract 2

The renewable PDL (poly-δ-decalactone) and mPEG-*b*-PDL (methoxy-poly(ethylene glycol)-*b*-PDL) based nanoemulsion (NE) bearing ketoconazole (KTZ) and eugenol (EUG) were developed and finally, KTZ–EUG–NE-based gel were investigated for topical delivery against *Candida albicans*.

## Graphical abstract

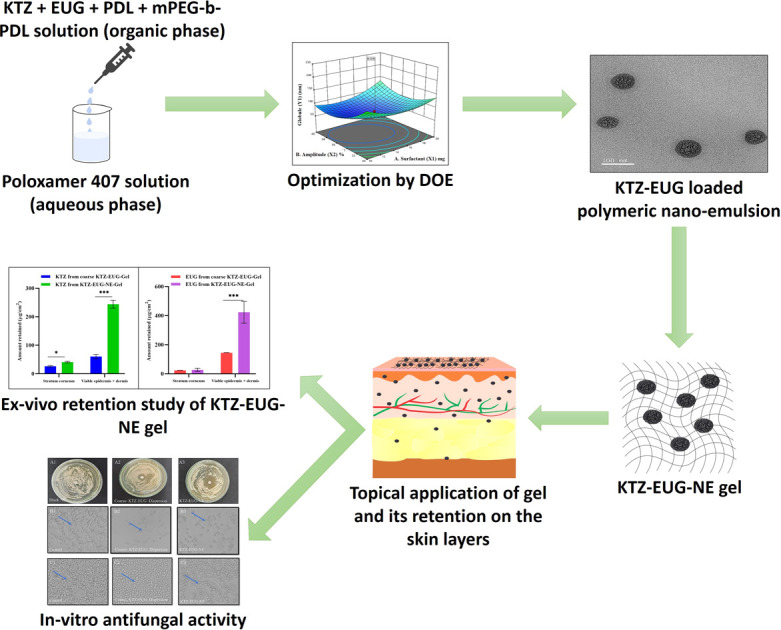

## 1. Introduction

Globally, the frequency of fungal infections is increasing continuously, and more than thirty-five million people are currently affected by superficial fungal infections.^1^ Among such, infections caused by the *Candida* species, especially *Candida albicans*, human fungal pathogens account for more of the reported worldwide deaths by fungi. It occurs at the superficial layer of the nails, skin, and hair (superficial mycosis) and can also spread to inner tissues.^2^ Various drugs (azoles, amphotericin B, allylamines, and echinocandins) have been employed for the treatment of *Candida albicans*. However, repeated use of such medicines leads to the development of resistance to single antifungal agents. Combination therapy is required to produce additive or synergistic effects at lower concentrations that can restrict the development of drug resistance against *Candida albicans*.^3^ Ketoconazole (KTZ) is a broad-spectrum imidazole drug with antifungal activity. Poor water solubility limits the antifungal potential of such drug, necessitating the use of drug carriers that can efficiently enhance the therapeutic potential of such agent.^4^ The existing conventional topical KTZ formulations are creams, gels, and lotions. However, all such formulations either have less skin penetration (creams and gels) or less contact time with the targeted area (lotions). Advanced nanotechnology-driven KTZ formulations, including nanostructured lipid carriers (NLCs), solid lipid nanoparticles (SLNs), poly(lactic-*co*-glycolic acid nanoparticles (PLGA NPs), and nanocomplexes, have been explored by researchers.^5–8^ KTZ has also been studied for combination approaches employing oils to produce nanoemulsions and microemulsion-based nanoemulgels.^9,10^ Eugenol (EUG), a phenolic aromatic oil belonging to the allylbenzene class, has been reported to have antifungal properties. The development of formulations containing such essential oils as bioactive agents has been restricted owing to their volatile nature, instability, and low bioavailability.^11,12^ The benefits of eugenol as a therapeutic agent have been studied through cubosomal and nanoemulsion-based approaches.^13,14^ Using KTZ and EUG as therapeutic agents can result in synergistic or potentiating effects. However, an effective drug nanocarrier is required to address the challenges of combining KTZ and eugenol for effective antifungal therapeutic applications.

Extensive research has been conducted on nanogel systems containing nanoemulsions (NEs). Nanoemulsions are isotropic and kinetically stable systems, in which the oil phases are stabilized by thin layers of emulsifying agents.^15,16^ The advancement in NEs has led to the development of polymeric nanoemulsions prepared from a polymer (PDL) as the viscous oil and a block copolymer (mPEG-*b*-PDL) as the surfactant. NEs prepared from such a viscous oily polymer exhibit physical stability and no sign of Ostwald ripening, as discussed in various literature.^17–20^ Polymers and copolymers have been explored as drug carriers (micelles and nanoemulsions).^21–23^

This study reports the first topical application of a nanoemulgel bearing a PDL-based nanoemulsion. Although the carrier system has been well studied to encapsulate hydrophobic moieties, mostly one drug, this study aims to incorporate dual drugs, *i.e.*, KTZ and EUG. The lipophilic and amorphous forms of PDL may enhance drug permeation through the skin and retention in the dermis or epidermis regions, thus providing an effective antifungal effect.^24,25^ The present investigation involves the quality by design (QbD)-driven development of a polymeric nanoemulsion using KTZ and EUG. The nanoemulgel was prepared and evaluated for topical antifungal applications by employing *ex vivo* skin permeation and retention studies. Finally, the *in vitro* antifungal activities were studied to define the effectiveness of the novel copolymer NE in topical applications.

## 2. Materials and methods

### Materials

PDL and mPEG-*b*-PDL were synthesized according to the procedure reported by Bansal *et al.* at Åbo Akademi University.^18,22^ Eugenol and dialysis membranes were purchased from Sigma-Aldrich Inc. (St. Louis, MO, U.S.A). KTZ, Carbopol-940, and Poloxamer-407 were purchased from Yarrow Chem Pvt. Ltd. (Maharashtra, India). Acetone, acetonitrile, and methanol were purchased from Merck Life Sciences Pvt. Ltd. (Maharashtra, India). All the materials used in the study had the highest purity grade >95%.

### Methodology

#### Preparation of the KTZ–EUG-NE

The preparation of the polymeric NE involves the nanoprecipitation method, followed by probe sonication. An oil-in-water (O/W) NE was prepared using PDL as a viscous oil, mPEG-*b*-PDL as surfactant-1, and Poloxamer 407 (P-407) as surfactant-2. In brief, a two-phase system was prepared, *i.e.*, the oil phase was prepared by employing KTZ, EUG, PDL, and mPEG-*b*-PDL in an acetone (oil phase), and P-407 was dissolved in water (aqueous phase). The NE was prepared through a two-stage process: first, dropwise addition of the oil phase to the aqueous phase with continuous stirring at 1000 rpm for 2 h. Next, a high-energy process, *i.e.*, probe sonication (Sonics Vibra Cell, Sonics & Materials Inc., Newton, USA), was used to generate uniform nano-sized droplets. The conditions used for sonication were a time of 15 min, 35% amplitude, controlled temperature, and a frequency of 05 s ON and 02 s OFF. A blank NE was also prepared using the same method but without the addition of drugs.^18^

#### Optimization of KTZ–EUG–NE

The QbD approach was used to develop the KTZ–EUG–NE. The quality target product profile (QTPP) defines the detailed description of drug product attributes that validate the necessary quality in terms of safety and efficacy. It aids in finding product critical quality attributes (CQAs) that conform to patient needs. Product and process development QTPPs were used to identify the CQAs. Acceptability ranges or limits can be established by CQAs to ensure the quality of the final product.

#### Optimization of KTZ–EUG–NE using the design of experiments (DOE)

To determine the impact of critical material attributes (CMAs) and critical process parameters (CPPs) (independent variables) on the CQAs (dependent variables) of topical KTZ–EUG–NE, the initial risk assessment and different CMAs and CPPs were selected based on previously reported literature and preliminary trial batches. The concentrations (mg) of the drug and surfactant were selected as CMAs, and the sonication amplitude (%) was selected as the CPP. These parameters were further evaluated by the classical design of experiments (DOE) technique using central composite design (CCD) to develop size-controlled reproducibility with the PDI, zeta potential, and optimum drug content.^26,27^ The design was selected on the basis of summary fit values (*P*-value and adjusted *R*^2^ values). A lack-of-fit test was used to evaluate the model's relevance, and a model with a non-significant variance difference over pure error variance was acceptable. The 3D response surface methodology (RSM) was represented by utilizing factors *vs.* response co-relations. It is also helpful for researching how different process factors interact. Three critical factors were selected with their two levels (−1 and +1 as low and high) to evaluate the effects of independent variables [*i.e.*, surfactant concentration (*A*), drug concentration (*B*), and amplitude (*C*)] on each dependent variable as responses, *i.e.*, globule size (*Y*_1_), polydispersity index (*Y*_2_), zeta potential (*Y*_3_), and drug content (*Y*_4_), as shown in Table 1.^28–30^

**Table 1:** Independent variables and dependent variables as responses in the central composite design

Independent variables as factors			Dependent variables as responses		
Critical factors	Altered levels		Response	Name	Units
	Low	High			
	−1	+1	*Y*_1_	Globule size	nm
*A*: Surfactant (mg)	10	20	*Y*_2_	PDI	Value
*B*: Drug (mg)	5	15	*Y*_3_	Zeta potential	mV
*C*: Amplitude (%)	30	40	*Y*_4_	Drug content	%

### Characterization of KTZ–EUG–NE

#### Droplet size, polydispersity index (PDI) and zeta potential

A Malvern Zetasizer (Nano ZS Malvern Analytical Ltd., UK) was used to determine the globule size, uniformity, and zeta potential of KTZ–EUG–NE. Briefly, the samples were diluted 10 times using Milli-Q water to acquire kilo counts per second (kcps) in the range of 200–500.^31^

#### Attenuated total reflectance (ATR) study

ATR (Bruker Alpha 2, USA) spectroscopy was employed to determine the interactions between excipients and drugs used in the NE. The change in the peaks in the ATR spectra denotes the interaction of the excipients used to prepare the formulations. The KTZ, EUG, PDL, mPEG-*b*-PDL, P-407, blank NE, and KTZ–EUG–NE samples weighing 3–5 mg were considered for the ATR study.^32^

#### Transmission electron microscopy (TEM)

TEM (JEM 2100 JEOL Ltd., Tokyo, Japan) was used to determine the morphology and globule size of the KTZ–EUG–NE. The sample was appropriately diluted, placed on a copper grid, and dried at room temperature. Further, the sample grid was placed in the TEM, and analysis was carried out at an energy of 120 kV for intervals of 120 s.^33^

#### Atomic force microscopy (AFM)

The surface roughness of the polymeric NE was measured using AFM (Asylum Research MFP-3D-BIO). The diluted KTZ–EUG–NE sample was spin-coated on a 2 cm^2^ glass slide and dried at 40 °C in a vacuum oven. The sample was placed under a microscope, and different images were taken.^18^

### Total drug content

The amount of KTZ and EUG in the NE was determined by employing the direct method of drug determination. Briefly, 1 mL of KTZ–EUG–NE was dissolved in 4 mL of methanol under probe sonication to break each globule. The sample was centrifuged, and the supernatant was filtered, diluted, and analyzed using the developed RP-HPLC method. The detection of KTZ and EUG was carried out using acetonitrile, methanol, and phosphate buffer (pH 5.5) as the mobile phase (50 : 30 : 20) and a Phenomenex Luna (C18, 5 μm, 240 × 4 mm) column as the stationary phase. The mobile phase was run at a flow rate of 1 mL min^−1^ and injection volumes were taken as 10 μL for quantifying each sample.^34^

#### *In vitro* drug release study

The dialysis bag approach was used to examine the *in vitro* drug release of KTZ–EUG–NE. The cellulose dialysis membrane (12 000 Da MWCO) was activated in EDTA solution before the release study. Briefly, a 1 mL sample (a coarse suspension of KTZ–EUG and KTZ–EUG–NE) was placed in a dialysis bag (DB) clipped at both ends. The DB was placed in an acceptor compartment (AC) that consisted of physiologically relevant media (phosphate buffer at pH 5.5 with 10% methanol). The process parameters employed were a speed of 100 rpm and a temperature of 37 ± 0.5 °C. At regular intervals of time (0.25, 0.5, 1, 2, 4, 8, 12, and 24 h), aliquots of 1 mL were withdrawn and replaced with the same media to maintain the sink conditions. To determine the amount of drug, all samples were filtered through 0.22 μm syringe filters and analyzed using the developed HPLC method.^34^ The experiment was performed in triplicate, and the data are presented as mean ± SD.

#### Release kinetics study

It is important to understand the underlying mechanism of drug release from polymeric NE. The *in vitro* drug release data was fitted to several release kinetic models, including zero-order, first-order, Higuchi, and Korsmeyer–Peppas. Higher regression coefficients (*R*^2^) and lower Akaike Information Criteria (AIC) were used to select the models.^35–37^

### Preparation and characterization of KTZ–EUG–NE–Gel

#### Preparation of KTZ–EUG–NE–Gel

The preparation of KTZ–EUG–NE–Gel involves the addition of Carbopol 940 to the KTZ–EUG–NE. Briefly, Carbopol 940 (1–2% w/v) was added to the KTZ–EUG–NE with continuous stirring for 2 h. The pH of KTZ–EUG–NE–Gel was adjusted to 6.5 by employing triethanolamine.^38^ The coarse KTZ–EUG–Gel was prepared using the same method; however, a coarse dispersion of KTZ–EUG was used instead of the NE.

### Evaluation of the gel

#### Physical examination and pH of the gel

The prepared KTZ–EUG–NE–Gel was visually examined for its color, appearance, and consistency. A calibrated digital pH meter (M/s Mettler Toledo, USA) was used to determine the pH of KTZ–EUG–NE–Gel. Briefly, 1 g of gel was dispersed in Milli-Q water to examine the pH using a washed electrode. The pH was determined thrice, and the values are represented as mean ± SD.^39^

#### Determination of the total drug content

The drug content of KTZ–EUG–NE–Gel was examined by adding 500 mg of gel to 30 mL of methanol. The mixture was sonicated for 15 min to disrupt the globule completely. Centrifugation was performed at 12 000 rpm for 30 min. The sample was further filtered, diluted, and examined for total drug content. The experiment was performed in triplicate, and the data were reported as mean ± SD.^40^

#### Rheological study

The viscosity of the prepared 1% w/v Carbopol KTZ–EUG–NE–Gel was determined using a rheometer (MCR 102, Anton Parr, Germany). A parallel plate geometry with a diameter gap of 0.20 mm was used in this study. The sample was placed between parallel plates, and a 0.1–100 s^−1^ shear rate was induced for 10 s frequency to check the rheological behavior. Finally, a graph was plotted between shear stress and shear strain.^32,41^

#### Determination of spreadability

The prepared 1% w/v Carbopol KTZ–EUG–NE–Gel was evaluated using a texture analyzer (TA-XT Plus, Japan). The compression test mode was used with a pre-test speed of 1.00 mm s^−1^, a test speed of 3.00 mm s^−1^, and a post-test speed of 10.00 mm s^−1^. A force of 100.0 g was employed to produce a strain of 10%. The sample was placed in the receiver compartment and recorded for firmness and adhesiveness.^34^

### *Ex vivo* skin permeation studies

The *ex vivo* permeation and skin retention studies were performed on fresh pig ear skin, and the detailed methodology is mentioned in the ESI (Sections 1.2 and 1.3).†

#### Cell cytotoxicity study

The biosafety of coarse KTZ–EUG, blank NE, and KTZ–EUG–NE was assessed in human keratinocyte (HaCaT) cells using the MTT assay. HaCaT cells were seeded in 96 well plates with a density of 5 × 10^−3^ cells per well. The cells were then treated with different concentrations (0.1, 0.5, 1, 5, 10, and 20 μg mL^−1^) of coarse KTZ–EUG, blank NE, and KTZ–EUG–NE and further incubated for 24 h at 37 °C. A 10 μL MTT solution (5 mg mL^−1^) was added to each well and incubated for 3 h at 37 °C. After that, 100 μL of DMSO was added to dissolve the formazan crystals, and the cells were analysed at 570 nm using a microplate reader (Synergy H1, BioTek).^42^

#### *In vitro* antifungal activities

The strain *C. albicans* ATCC 90028 was procured from the Institute of Microbial Technology, Chandigarh, India. The strain was maintained on yeast extract, peptone, and dextrose agar plates and stored at 4 °C. A single isolated colony of *C. albicans* was inoculated in YPD broth and incubated for 24 h at 30 °C. The disk diffusion assay (DDA), the effect of the formulations on planktonic growth, yeast to hyphal morphogenesis, and *C. albicans* film formation were studied^34^ and the detailed methodology is mentioned in the ESI (Section 1.5).†

#### Irritation study by HET-CAM assay

The toxicity of blank NE and KTZ–EUG–NE was assessed using the hen's egg test-chorioallantoic membrane (HET-CAM) assay. The experiment was conducted in a laminar airflow hood using sterilized equipment. Freshly fertile eggs were obtained from the Central Hatchery Center in Kolhapur, India, and were incubated at 37 °C and 60% humidity for five days. After incubation, the eggs' outer surface was treated using 70% isopropyl alcohol to open the broader end, and the inner vitelline membrane was extracted, revealing the CAM. The exposed CAM was investigated using 0.9% NaCl as a negative control and 300 μL of 0.1 M NaOH as a positive control to determine the hemorrhage and coagulation. To investigate the irritant effect, the CAM was treated using 10 μg mL^−1^ of blank NE and KTZ–EUG–NE. The opened portion of the eggs was sealed, and the eggs were further incubated for 24 h. The CAM was investigated for signs of irritation (hemorrhage and coagulation), and images were taken.^43^

### Statistical data

Most experiments were performed in triplicate, and the resulting data are presented as mean ± standard deviation (SD). The analysis of binary data was conducted using Design Expert (Version 13), DD solver, and Graph Pad Prism 9.1.0 (GraphPad software, USA). A *P*-value < 0.05 was considered significant in the whole experiment.

## 3. Results and discussion

### Synthesis and characterization of polymers

The synthesis and purification of PDL and mPEG-*b*-PDL were confirmed by ^1^H NMR (ESI Fig. S1†), and the results were in accordance with previously reported values.^22^ According to NMR, the molecular weight of the homopolymer was calculated to be 5.2 K using proton integrals at 4.9, 5.1, and 7.3 ppm. Similarly, the molecular weight of the copolymer was calculated to be 8.7 K using proton integrals at 0.9, 3.4 and 4.9 ppm.

### Experimental design for developing KTZ–EUG–NE

**Fig. 1:**
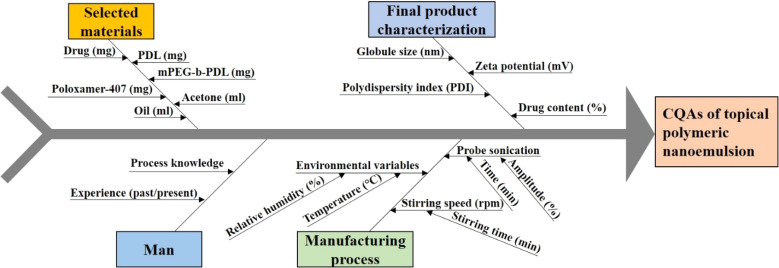
Ishikawa fish-bone diagram to showcase the potential cause-and-effect of polymeric nanoemulsion.

The CQA and CMA governing the quality target product profile are depicted in Fig. 1. The CQAs considered in the study were taken on the basis of the literature and initial trials. The concentrations of drugs and surfactant were selected as CMAs, whereas the sonication amplitude was selected as the CPP to generate a polymeric NE using the nano-precipitation method. The QTPP ensures the safety, efficacy, and applicability of the finished product. ESI Table S1† explains the main QTPP parameters used to generate the polymeric NE.

### DOE for the optimization of KTZ–EUG–NE

The CCD design was used to optimize KTZ–EUG–NE by employing twenty runs (Table 2). Variations in the globule size (nm), polydispersity index (PDI), zeta potential (mV), and % drug content were examined by response surface methodology that explains the correlations among each factor in the design of the polymeric NE. All data were statistically analyzed and the best-fit model was determined for the independent variables of the polymeric NE.^44,45^ After 20 successful runs, all responses (*Y*_1_, *Y*_2_, *Y*_3_, and *Y*_4_) exhibited a quadratic model with significant *p* values and higher *R*^2^ values. The values of lack of fit (*P* > 0.05), predicted *R*^2^, and adequate precision were found to be in good agreement, demonstrating that the models are reliable and can be used to navigate the design space.

**Table 2:** Central composite design (CCD) based experimental trials

Run	Surfactant (*A*) (mg)	Amplitude (*B*) (%)	Drug (*C*) (mg)	Globule size (nm) (*Y*_1_)	PDI (*Y*_2_)	Zeta potential (*Y*_3_) (mV)	Drug content (*Y*_4_) (%)
1	15	35	10	69.07	0.2	−3.59	83.99
2	15	35	10	77.05	0.2	−2.3	86.69
3	20	40	5	157.7	0.25	1.48	74.41
4	20	30	15	233.1	0.4	−1.11	91.03
5	10	40	5	129	0.15	−3.3	69.73
6	15	35	10	62.19	0.16	−3.5	86.3
7	15	35	1.5	80.98	0.24	−0.766	59.68
8	15	35	10	68	0.18	−2.85	85.35
9	23.4	35	10	137	0.3	1.04	87.04
10	15	26.5	10	194	0.28	−9.78	76.37
11	15	35	10	62	0.16	−4.2	83.47
12	6.5	35	10	142	0.3	−4.9	77.83
13	15	43.4	10	130.1	0.23	−4.9	88.53
14	10	40	15	102.2	0.4	−8.25	88.48
15	20	40	15	179	0.35	−0.361	90.01
16	20	30	5	102	0.363	−0.335	70.23
17	15	35	18.4	173	0.537	−3.43	92.03
18	15	35	10	60.31	0.18	−2.72	84.67
19	10	30	15	215.4	0.464	−6.2	84.3
20	10	30	5	131	0.3	−5.25	64.29

The ANOVA table was obtained for each response, explaining the important and significant factors that must be considered during the development of KTZ–EUG–NE. Each response was explained for its correlation with independent factors using coded equations. The average globule size of KTZ–EUG–NE was found to be in the range of 60.31 ± 0.04 to 233.1 ± 0.43 nm. The significant factors obtained were *B*, *C*, *BC*, *A*^2^, *B*^2^, and *C*^2^, as given in ESI Table S2.† The coded equations obtained for each response explained the correlations among these factor to generate the desired responses.

1 *Y*_1_ (globule size) = 66.16 + 6.28*A* − 16.19*B* + 26.71*C* + 14.60*AB* + 11.85*AC* − 27.63*BC* + 27.67*A*^2^ + 35.64*B*^2^ + 23.24*C*^2^

**Fig. 2:**
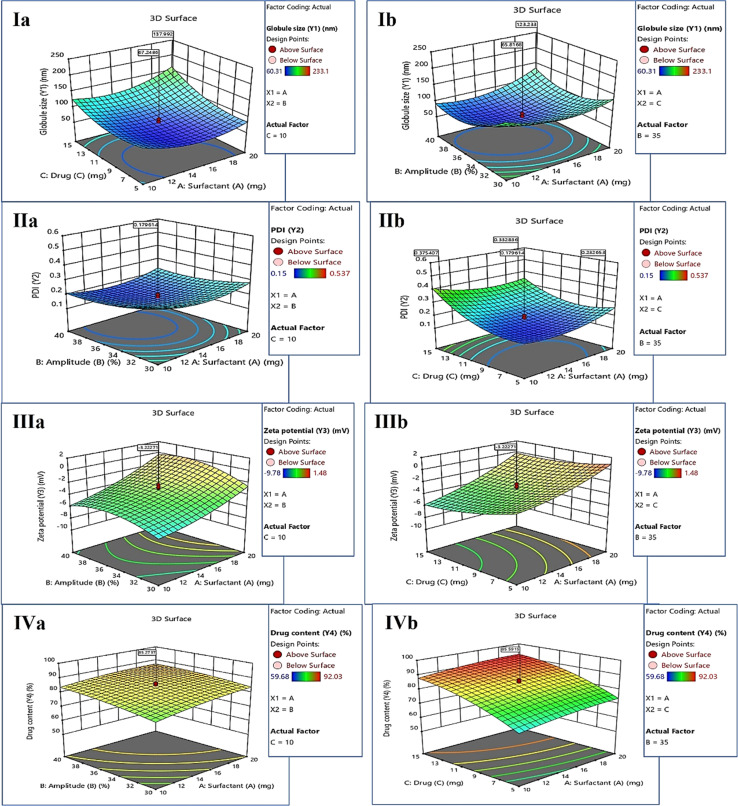
3D representation of each response with its crucial factor. (Ia) Globule size (*Y*_1_) *vs.* surfactant concentration (*A*) and drug concentration (*C*) and (Ib) globule size (*Y*_1_) *vs.* surfactant concentration (*A*) and sonication amplitude (*B*). (IIa) PDI (*Y*_2_) *vs.* surfactant concentration (*A*) and sonication amplitude (*B*) and (IIb) PDI (*Y*_2_) *vs.* surfactant concentration (*A*) and drug concentration (*C*). (IIIa) Zeta potential (*Y*_3_) *vs.* surfactant concentration (*A*) and sonication amplitude (*B*) and (IIIb) zeta potential (*Y*_3_) *vs.* surfactant concentration (*A*) and drug concentration (*C*). (IVa) Drug content (*Y*_4_) *vs.* surfactant concentration (*A*) and sonication amplitude (*B*) and (IVb) drug content (*Y*_4_) *vs.* surfactant concentration (*A*) and drug concentration (*C*).

The positive sign in the equations (eqn (1)–(4)) denotes a direct correlation between the response and the factors; however, the negative sign indicates an indirect or negative correlation. Significant terms were considered according to the coded equation to justify the correlation. An inverse correlation was found between the globule size (*Y*_1_) and amplitude (*B*) owing to the presence of sonic waves for the reduction of globule size (eqn (1)). However, the concentration of drugs showed a positive correlation with globule size, governing the nanoprecipitation of free drug when taken at higher concentrations. The increase in globule size was also governed by the square effect of *A*, *B*, and *C*, as shown in Table S2.† The correlation was represented by employing 3D surface plots for the globule size as a response. As shown in Fig. 2(Ia), a lower concentration of the drug decreases the globule size because of the solubility differences in the drugs. The increase in surfactant concentration decreases the globule size, as can be visualized in Fig. 2(Ib), which also explains the negative correlation between the globule size and amplitude of sonication.

The average polydispersity index (PDI) of KTZ–EUG–NE after twenty runs was found to be in the range of 0.15 ± 0.03 to 0.537 ± 0.23. The significant factors examined by ANOVA were *B*, *C*, *AC*, *A*^2^, *B*^2^, and *C*^2^, as given in ESI Table S3.† The coded equation generated to study the correlation between PDI responses and factors is given below (eqn (2))

2 *Y*_2_ (PDI) = 0.1796 + 0.0036*A* − 0.0338*B* + 0.0769*C* + 0.0064*AB* − 0.0346*AC* + 0.0186*BC* + 0.0450*A*^2^ + 0.0290*B*^2^ + 0.0762*C*^2^

According to the equation, an increase in the sonication amplitude decreases the PDI value, producing uniform globules. However, a positive correlation was observed between the additive effect of amplitude (*B*^2^) and PDI (*Y*_2_) values owing to coalescence resulting from the decreased globule size. The concentration of KTZ and EUG was found to be another significant factor that directly correlated with an increase in the PDI. The increase in the PDI value was also governed by an increase in the concentration of surfactant, as depicted in the equation and 3D response plots of Fig. 2(IIa) and (IIb).

The average zeta potential (ZP) of KTZ–EUG–NE was found to be in the range of −9.78 to 1.48 mV. For ZP, the response significant terms considered were *A*, *C*, and *B*^2^, as given in ESI Table S4.†

3 *Y*_3_ (zeta potential) = −3.22 + 2.39*A* + 0.7814*B* − 0.9516*C* + 0.3330*AB* + 0.4105*AC* − 0.6332*BC* + 0.6386*A*^2^ − 1.27*B*^2^ + 0.5792*C*^2^

An inverse correlation was found between the zeta potential (*Y*_3_) and surfactant-2 (*A*) owing to the amphiphilic nature that was responsible for the neutralization of the zeta potential (eqn (3)). The equation also explained the negative correlation between the zeta potential and the concentration of the drugs employed in the study. The correlation was visualized using 3D response surface plots, as shown in the plots of Fig. 2(IIIa) and (IIIb).

The average KTZ content from KTZ–EUG–NE was found to be in the range of 59.68 ± 0.56% to 92.03 ± 0.37%. The factors that significantly affected the KTZ content of KTZ–EUG–NE were found to be *A*, *B*, *C*, and *C*^2^, as shown in ESI Table S5.† According to eqn (4), an increase in the surfactant and drug concentration increases the drug content of the NE owing to an increase in the solubility of KTZ in the presence of surfactant. The higher amplitude also increases the drug content of the NE because of the high energy-driven entrapment of KTZ in the polymeric NE. The correlation of each factor can be visualized using the 3D surface plots in Fig. 2(IVa) and (IVb).

4 *Y*_4_ (% drug content) = 85.11 + 2.52*A* + 2.43*B* + 9.49*C* − 0.8075*AB* − 0.2950*AC* − 0.8075*BC* − 1.12*A*^2^ − 1.12*B*^2^ − 3.45*C*^2^

### Optimization of KTZ–EUG–NE

Statistical optimization was conducted by employing the desirability function (*D*). To achieve the QTPP for the polymeric NE, optimization was carried out using solutions from a design expert. The formulations were prepared according to the optimized protocol (CPP), and responses were recorded (CQA). The predicted values for each response *Y*_1_–*Y*_4_ were recorded and compared with experimental values to further calculate the prediction error percentage, which was found to be <20% (Table 3).
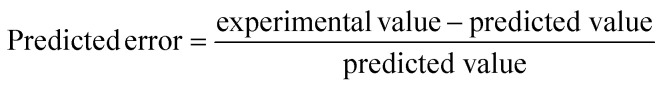


**Table 3:** Predicted error for KTZ–EUG–NE

*A* : *B* : *C*	Response variables	Experimental value	Predicted value	Predicted error (%)
Surfactant (*A*), 15 mg	Globule size (*Y*_1_)	68.91	66.15	4.17
Amplitude (*B*), 35%	PDI (*Y*_2_)	0.191	0.17	12.35
Drug (*C*), 10 mg	Zeta potential (*Y*_3_)	−3.36	−3.2	5.1
	% drug content (*Y*_4_)	84.33	85.10	3.1

### Physicochemical characterization of KTZ–EUG–NE

#### Droplet size, polydispersity index, and zeta potential (ZP)

**Fig. 3:**
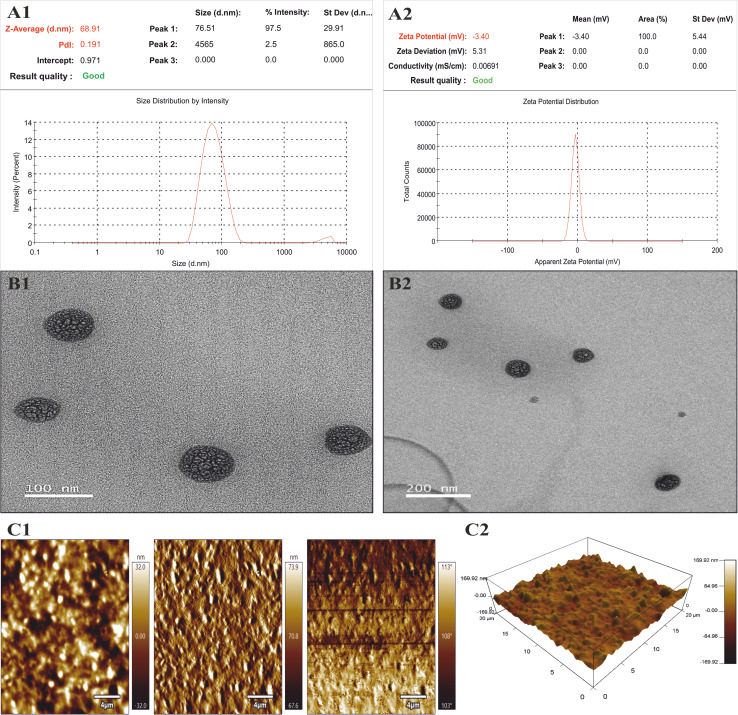
(A1 and A2) Globule size and zeta potential of the optimized KTZ–EUG–NE. (B1 and B2) TEM images of KTZ–EUG–NE. (C1 and C2) Topography of KTZ–EUG–NE confirmed through AFM.

The globule size of the optimized KTZ–EUG–NE was found to be 68.91 ± 3.4 nm, as determined by the dynamic light scattering principle using a Zetasizer. The globule size distribution also showed a narrow polydispersity index of 0.191 ± 0.05, as shown in Fig. 3(A1). The zeta potential of the optimized formulation was found to be −3.40 ± 0.19 mV, explaining that the stability of the NE was charge independent, as shown in Fig. 3(A2). This was the steric hindrance produced due to the coating of mPEG-*b*-PDL, which provides stability to the NE.

#### Attenuated total reflectance (ATR) study

The ATR study depicted the interaction among pure KTZ, EUG, PDL, mPEG-*b*-PDL, and P-407 used in the development of blank NE and KTZ–EUG–NE, as shown in ESI Fig. S2.† KTZ showed a characteristic peak at 1647.26 cm^−1^ for C=O stretching, 1031.95 cm^−1^ for the stretching of the aliphatic ether group (C–O), 1244.13 cm^−1^ for the stretching of the cyclic ether (C–O), 1510 cm^−1^ for aromatic asymmetric stretching (C=C), 1584 cm^−1^ for aromatic symmetric stretching (C=C), and 3119 cm^−1^ for (=C–H stretching). EUG showed characteristic peaks at 3521 cm^−1^ (OH stretching), 1365 cm^−1^ (isopropyl group) and 1516 cm^−1^ (C=C), which was similar to a previously reported study.^46^ The polymer (PDL) had characteristic peaks at 2928.10 cm^−1^ (C–H) and 1166.71 cm^−1^ (C–O). Peaks at 2877.01 cm^−1^ (C–H stretching), 1729.65 cm^−1^ (C=O), and 1102.11 cm^−1^ (aliphatic O–CH_2_–CH_2_) were observed for mPEG-*b*-PDL. P-407 had characteristic peaks at 2882.18 cm^−1^ (C–H) and 1353.30 cm^−1^ (CH–CH_3_). In the case of blank NE, some distinctive peaks of the excipients were observed at 1108.22 cm^−1^, 1730.74 cm^−1^ (C=O), 2876.66 cm^−1^ (C–H stretching), and 1342.18 cm^−1^ (CH–CH_3_), except for those of the drug and oil. The spectrum of KTZ–EUG–NE showed similar peaks to the blank spectrum; however, the peak at 1520 cm^−1^ may be governed by the presence of the aromatic asymmetric stretching (C=C) of KTZ or EUG. The observed findings may illustrate the entrapment of KTZ and EUZ inside the polymeric PDL.^47^

#### Transmission electron microscopy (TEM) and atomic force microscopy (AFM)

The TEM images depicted the morphology of the prepared KTZ–EUG–NE (Fig. 3(B1) and (B2)). The NE possessed nanosized spherical shaped globules. The spherical shape aids the NE in squeezing from the pores of the skin, thereby promoting maximum skin permeability to achieve topical applications. The topography of KTZ–EUG–NE is depicted in Fig. 3(C1) and (C2). The 2D and 3D plots from the AFM analysis showed the rough surface of the polymeric NE. The adsorption of mPEG-*b*-PDL on the PDL might result in a rough surface.

#### Total drug content and *in vitro* drug release study of KTZ–EUG–NE

**Fig. 4:**
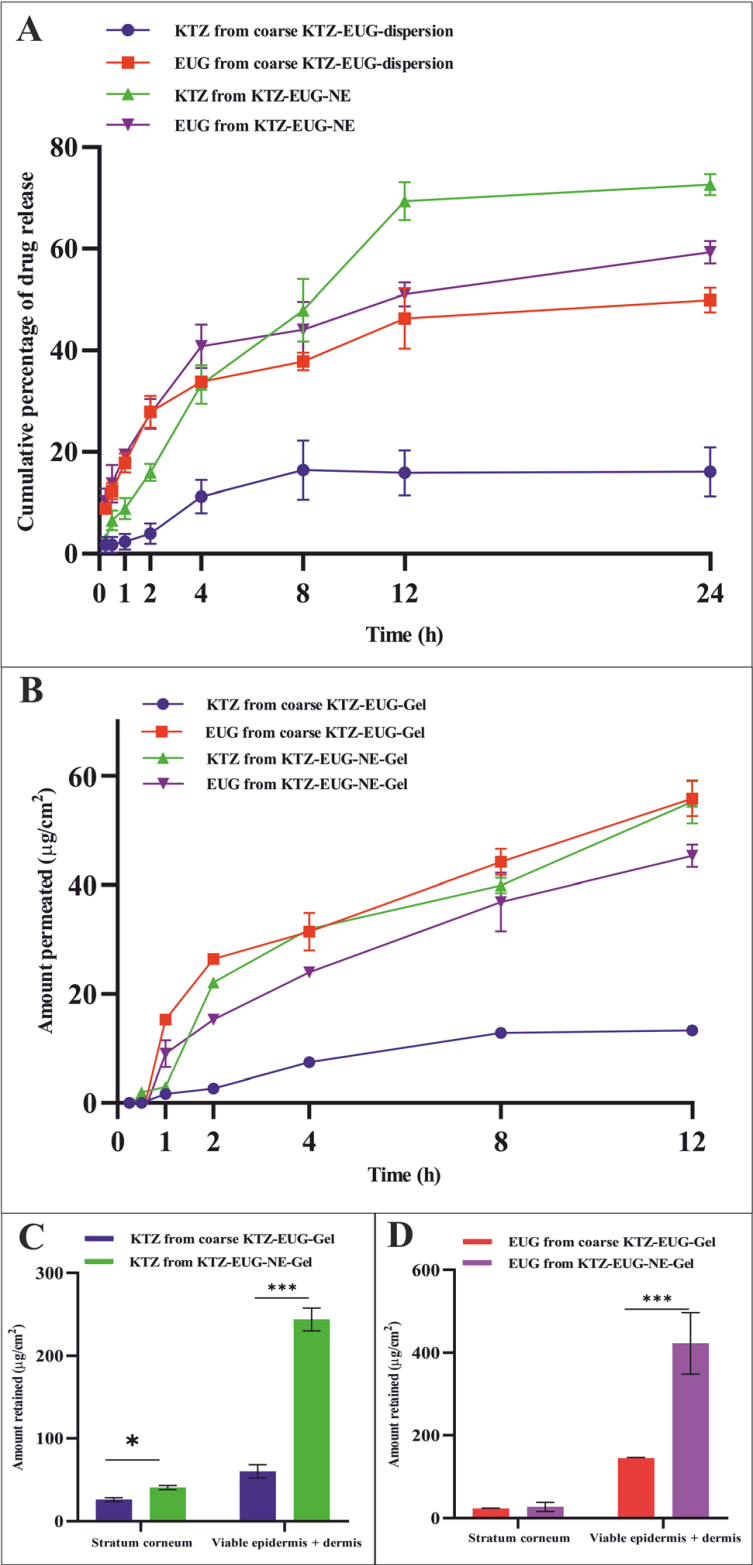
(A) *In vitro* drug release study of KTZ and EUG from the coarse KTZ–EUG dispersion and KTZ–EUG–NE, (B) *ex vivo* skin permeation study of KTZ and EUG from coarse KTZ–EUG–Gel and KTZ–EUG–NE–Gel, and *ex vivo* skin retention study illustrating (C) KTZ retained from coarse KTZ–EUG–Gel and KTZ–EUG–NE–Gel and (D) EUG retained from coarse KTZ–EUG–Gel and KTZ–EUG–NE–Gel in the stratum corneum, viable epidermis, and dermis region of the skin (*** represents *p* < 0.001).

After successful optimization, the average KTZ and EUG contents in KTZ–EUG–NE were found to be 84.33 ± 0.25% and 85.95 ± 0.05%, respectively. The coarse KTZ–EUG dispersion and KTZ–EUG–NE were evaluated for *in vitro* drug release and analyzed for the cumulative amount of KTZ and EUG released from the NE within 24 h, as shown in Fig. 4(A). KTZ is a poorly water-soluble drug showing only 16.09 ± 4.78% release from the coarse KTZ–EUG dispersion. However, the release performance of KTZ from KTZ–EUG–NE showed a drastic change with 72.65 ± 2.06% release at 24 h owing to the increase in aqueous solubility. In the case of EUG, 49.89 ± 2.38% release was observed from the coarse KTZ–EUG dispersion in contrast to 59.28 ± 2.23% from KTZ–EUG–NE (after 24 h). As explained by Pyrhönen *et al.*, nanoemulsions prepared using PDL and mPEG-*b*-PDL are known to enhance the solubility of hydrophobic drugs.^19^

The cumulative percentage of drug release data was fitted into different release kinetic models, *viz.*, zero-order, first-order, Higuchi, and Korsmeyer–Peppas. The best-fit model was determined on the basis of a higher regression coefficient (*R*^2^) and lower AIC values. The data for KTZ and EUG release from KTZ–EUG–NE were best fitted to the Korsmeyer–Peppas model with the highest *R*^2^ values of 0.965 and 0.964, respectively. Moreover, the Korsmeyer–Peppas model showed the lowest AIC values of 45.12 and 39.15 for KTZ and EUG, respectively, as depicted in ESI Table S6.† The release exponent values were found to be in the range of 0.45–0.89, explaining that the NE follows anomalous (non-Fickian) diffusion of drug release.

### Characterization of KTZ–EUG–NE–Gel

The NE-loaded gel was evaluated for physical properties (color, texture, pH, and homogeneity). NE–Gel prepared using a 1% w/v Carbopol concentration possessed a whitish appearance with greater homogeneity and no indications of grittiness or lumps (ESI Fig. S3†). However, NE–Gel prepared using a 2% w/v Carbopol concentration showed a clumsy appearance and was thus excluded from further evaluation. The prepared NE–Gel possessed a pH value of 6.2 ± 0.04, which is essential for avoiding irritation and ease of topical application against fungal infection.^48^ The content of KTZ and EUG in 1% w/v Carbopol KTZ–EUG–NE–Gel was found to be 81.32 ± 0.23% and 83.24 ± 0.95%, respectively. The observed concentration of drugs explained the uniformity of the NE-loaded gel.

#### Spreadability study

The firmness and work of shear determined for the sample were found to be 534.67 ± 65.2 g and 399.37 ± 53.2 g s, respectively (ESI Fig. S4†). The observed values adequately explained the hydrogel's optimum spreadability, as Kumar *et al.* explained.^35^

#### Rheological study

Rheology is the study of resistance to the flow of liquid and establishes a correlation between shear stress and shear rate. ESI Fig. S5B† explains the deformation of the hydrogel when the applied shear rate from 0.09 (s^−1^) to 100 (s^−1^) linearly produces shear stress. The generated shear stress decreases the viscosity of KTZ–EUG–NE–Gel owing to the deformation of the gelling matrix (ESI Fig. S5A†). The shear thinning mechanism of KTZ–EUG–NE–Gel was governed by the loss of interaction among the Carbopol chains. The viscosity of the optimized 1% w/v Carbopol 940 containing KTZ–EUG–Gel was found to be 10 942 ± 11.92 mPa s, which depicted the viscous nature of the prepared gel.

### *Ex vivo* skin permeation and retention studies

The skin permeability of KTZ–EUG–NE was assessed using an *ex vivo* permeation study (Fig. 4(B)). The amount of KTZ permeated through the 3.14 cm^2^ area of the skin was found to be 18.44 ± 2.97 μg and 71.28 ± 1.54 μg from coarse KTZ–EUG–Gel and KTZ–EUG–NE–Gel, respectively. A 5-fold higher transdermal flux (*J*_ss_) accounted for KTZ–EUG–NE–Gel (1.819 μg h^−1^ cm^−2^) compared to coarse KTZ–Gel (0.371 μg h^−1^ cm^−2^) might be due to the nanosize range and lipophilic nature of the PDL, which helps in drug penetration. The values of the permeability coefficient (*K*_p_) for KTZ–EUG–NE–Gel and coarse KTZ–EUG–Gel were found to be 0.047 × 10^−3^ h^−1^ cm^−2^ and 0.4 × 10^−2^ h^−1^ cm^−2^, respectively.

In addition, the lipophilic nature of EUG helps to attain greater permeation, as reported in various articles.^49^ The study depicted *J*_ss_ values for EUG permeated from coarse KTZ–EUG–Gel and KTZ–EUG–NE–Gel as 1.41 μg h^−1^ cm^−2^ and 3.22 μg h^−1^ cm^−2^, respectively, suggesting a 2-fold higher permeation. The *K*_p_ values for coarse KTZ–EUG–Gel and KTZ–EUG–NE–Gel were determined to be 0.23 × 10^−2^ h^−1^ cm^−2^ and 0.5 × 10^−2^ h^−1^ cm^−2^, respectively.

After 12 h of application on the skin, KTZ–EUG–NE–Gel was evaluated for *ex vivo* retention on the superficial layer of the skin (the stratum corneum and the viable epidermis or dermis) (Fig. 4(C) and (D)). The poorly water-soluble KTZ when loaded in the polymeric NE exhibited 1.59 times higher retention at the stratum corneum and 4.03 times higher retention in the viable epidermis and dermis layers in contrast to coarse KTZ–EUG–Gel. However, EUG, owing to its lipophilic nature, restricts the retention in the superficial layer when applied in the form of coarse KTZ–EUG–Gel. It was the polymeric NE that retained a greater amount (2.93-fold) of EUG when compared with coarse KTZ–EUG–Gel in the viable epidermis or dermis. The findings from the study explain that the presence of the drug in the upper layers of the skin could provide a better treatment of superficial mycoses (*Candida albicans*).

The *Candida albicans* infection mainly occurs in the top layer of the skin, *i.e.*, the epidermis, as reported in various studies.^50^ After the successful completion of an *ex vivo* permeation study, higher fluorescence was observed in the epidermis after 12 h, as shown in ESI Fig. S6.† The layer is generally lipophilic in nature, and owing to the lipophilic character of PDL, lipid depots may form that can retain the drug for a longer time. In contrast to the brightfield view, the fluorescent view depicted the presence of FITC in the epidermis region. However, a tiny fraction of fluorescence was observed in the dermis region of the skin, which may be due to the hydrophilic properties of the dermis layer.^51^ The amorphous characteristics of the PDL may govern the permeation of FITC into the dermis layer; thus, a lower fraction was detected.

#### Cell cytotoxicity study

**Fig. 5:**
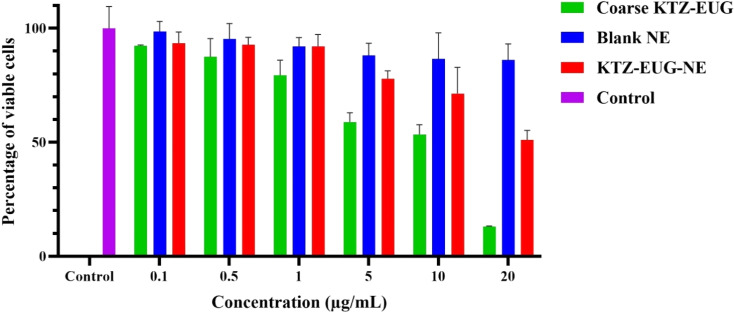
Cytotoxicity profile of coarse KTZ–EUG, blank NE, and KTZ–EUG–NE towards HaCaT cells at 0.1–20 μg mL^−1^ concentrations.

The cell viability of coarse KTZ–EUG, blank NE, and KTZ–EUG–NEKTZ–EUG–NE was determined in HaCaT cells in a concentration range of 0.1–20 μg mL^−1^. A higher (>70%) viability of HaCaT cells was reported to be non-cytotoxic,^42^ and thus coarse KTZ–EUG (up to 1 μg mL^−1^), blank NE (up to 20 μg mL^−1^), and KTZ–EUG–NE (up to 10 μg mL^−1^) may be considered non-cytotoxic (Fig. 5). KTZ–EUG–NE was found to be safer for HaCaT cells with only 28.65 ± 11.52% cytotoxicity at 10 μg mL^−1^. In contrast, coarse KTZ–EUG exhibited higher cytotoxicity (46.55 ± 4.30%) at a similar concentration. The lower cytotoxicity of KTZ–EUG–NE may be due to drug encapsulation in the nanoemulsion, which resulted in minimum cellular damage.^42^ This study revealed that the polymeric nanoemulsion (blank NE and KTZ–EUG–NE) was safer than coarse KTZ–EUG, indicating the potential of PDL and mPEG-*b*-PDL for topical skin applications.

#### Antifungal activities

**Fig. 6:**
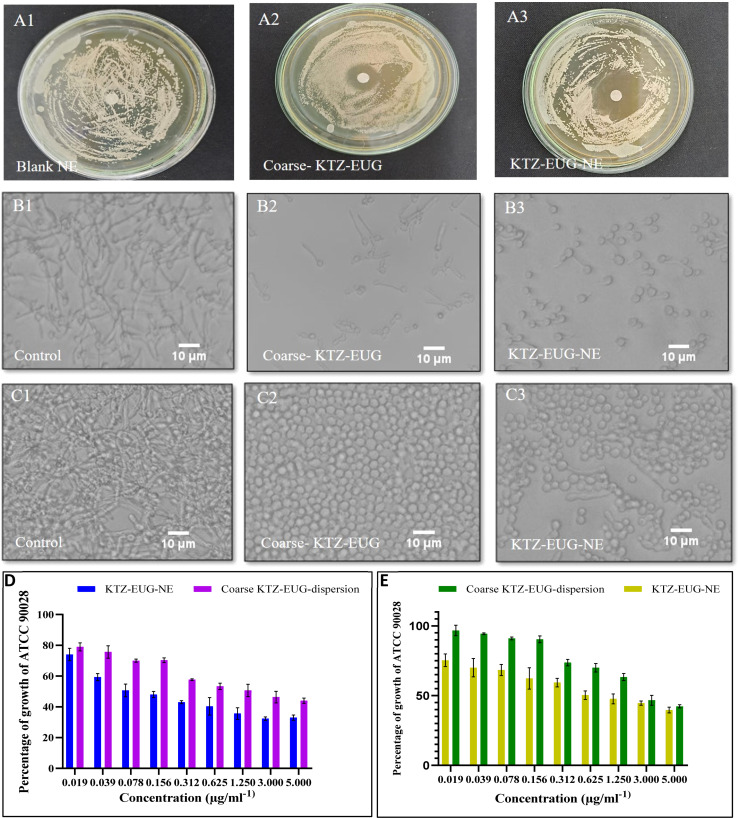
*In vitro* antifungal activities of the coarse KTZ–EUG dispersion and KTZ–EUG–NE illustrating (A1–A3) the zone of inhibition, (B1–B3) yeast to hyphal morphogenesis, (C1–C3) antibiofilm activity, (D) planktonic growth, and (E) antibiofilm.

The blank NE showed no sign of a zone of inhibition, as shown in Fig. 6(A1). However, KTZ–EUG–NE and coarse KTZ–EUG dispersions exhibited significant zones for inhibition owing to the antifungal activities of KTZ and EUG at different concentrations. 2–2.5 mm (Fig. 6(A2)) and 3–3.5 mm (Fig. 6(A3)) zones of inhibition were observed for the coarse KTZ–EUG dispersion and KTZ–EUG–NE, respectively. The higher inhibition of KTZ–EUG–NE may be attributed to the lipophilic nature of the PDL and nanosize range, which enhance the solubility and permeation of tested drugs.^52^

#### Planktonic growth

The effect of KTZ–EUG–NE on the planktonic growth of *C. albicans* was investigated using the *C. albicans* ATCC 90028 strain. As shown in Fig. 6(D), the planktonic growth of *C. albicans* was found to be inhibited in a concentration-dependent manner, where 19.23 times lower MIC_50_ was observed for KTZ–EUG–NE (0.156 μg mL^−1^) than for coarse KTZ–EUG dispersion (3 μg mL^−1^). The higher activity of NEs may be attributed to their nanosize range, which enhances the solubility and permeation of tested drugs.^52^ The amorphous and lipophilic nature of PDL may also be governed to produce such an effect by an increase in the permeation of antifungal drugs, as explained by Bansal *et al.*^21^

#### Formulation interfering with yeast to hyphal form conversion in *C. albicans*

The filamentous morphology can be observed in *C. albicans* when germ tubes from yeast and mold-like growth of branching hyphae arise. Such yeast–hyphal conversion is a virulence factor present in *C. albicans*. These morphological forms play essential roles in disease progression, invasion, adhesion, and host tissue damage. Targeting these morphological transitions in *C. albicans* could improve the treatment efficiency.^53^ The coarse KTZ–EUG dispersion and KTZ–EUG–NE inhibit the development of hyphae formation. Inhibition in transition was observed at 0.62 μg mL^−1^ and 0.156 μg mL^−1^ concentrations of coarse KTZ–EUG dispersion and KTZ–EUG–NE, respectively. The blank NE was found to be inactive in inhibiting yeast to hyphal conversion. Fig. 6(B) demonstrates the contrasting effect of KTZ–EUG–NE and coarse KTZ–EUG dispersion on yeast to hyphal formation conversion. The blue arrows in Fig. 6(B1) depict yeast to hyphal (Y–H) formation (control group). However, at 0.31 μg mL^−1^ concentration, no inhibition (Y–H) was observed when treated with the KTZ–EUG dispersion. Treatment with KTZ–EUG–NE at the same concentration of 0.31 μg mL^−1^ inhibited Y–H formation, as only yeast cells were observed in Fig. 6(B3).

#### Effect of the formulation on *Candida albicans* biofilm formation

Biofilm formation is a virulence factor that provides a protective mechanism against antifungal drugs and host immune defenses. The coarse KTZ–EUG dispersion and KTZ–EUG–NE inhibited the development of the biofilm in a concentration-dependent manner (Fig. 6(E)). However, KTZ–EUG–NE exhibited higher activities at lower concentrations (4 times) than the coarse KTZ–EUG dispersion. In contrast to the control and coarse KTZ–EUG dispersion treated groups at 0.31 μg mL^−1^, only yeast and some hyphal growth was observed, confirming the *Candida albicans* biofilm formation activity of KTZ–EUG–NE. Anti-biofilm activities were confirmed through inverted microscopy and XXT metabolic assay.^54,55^

#### Irritation study by HET-CAM assay

The HET-CAM assay has been widely used as an irritant model for ocular studies. The assay has also found its role as an irritant model to investigate the irritation effect of topical formulations as reported in various studies.^43,56–59^ The same was used to investigate the irritant properties of blank NE and KTZ–EUG–NE. The positive control after treatment with 0.1 M NaOH depicted the presence of hemorrhage. However, the negative control, blank NE, and KTZ–EUG–NE resulted in increased normal vascular density, as shown in ESI Fig. S8.† The findings depicted the blank NE and KTZ–EUG–NE as safer, with no significant sign of hemorrhage or coagulation as observed in the positive control. The non-irritant effect from the HET-CAM assay strengthens the safety profile of KTZ–EUG–NE as observed using the HaCaT cell cytotoxicity study.

## 4. Conclusion

Every year, approximately 150 million people are infected with *Candida albicans*. The drug combination of KTZ and EUG was successfully investigated using a newly explored polymeric NE. Renewable PDL and mPEG-*b*-PDL based NEs were produced using a central composite design through Design Expert® (version 13) software. The concentration of surfactant, drugs, and sonication amplitude were found to be crucial factors in the development of KTZ–EUG–NE. The QbD-driven development of the NE results in globule size < 100 nm, PDI < 0.3, and a total drug content for KTZ and EUZ of 80–85%. The rough-surfaced spherical NE was governed by the adsorption of mPEG-*b*-PDL on the globule, as confirmed by AFM and TEM analysis. The *in vitro* drug release study results depicted an initial fast release, followed by a sustained release effect with a non-Fickian diffusion-release model for KTZ and EUG. A 1% w/v gel was successfully developed and evaluated for physical characteristics, pH, and drug content. The optimal spreadability and viscosity of the KTZ–EUG–NE-based nanoemulgel explained the ease of topical application. The higher retention of KTZ and EUG from KTZ–EUG–NE onto the skin, mainly in the epidermis layer, concluded the localization of drugs to facilitate antifungal activity at the disease site. KTZ–EUG–NE was found to be effective in inhibiting planktonic growth and yeast to hyphal morphogenesis at significantly lower concentrations than the coarse KTZ–EUG dispersion. The inhibition of biofilm formation by KTZ–EUG–NE explains the importance of developing KTZ–EUG–NE. Moreover, the *in vitro* cell line and irritation studies depicted the safety and non-irritant nature of PDL and mPEG-*b*-PDL-based NEs for topical applications. The overall findings showed the potential of KTZ and EUG based polymeric nanoemulsions for topical delivery of antifungal agents. Further preclinical studies on dermatophytosis animal models are required to evaluate the antifungal efficacy that will strengthen the role of such biocompatible PDL and mPEG-*b*-PDL-based NEs.

## Conflicts of interest

The authors report no conflict of interest related to the manuscript.

## Supplementary Material

NA-006-D4NA00176A-s001

## Data Availability

The data supporting this article have been included as part of the ESI.†

## References

[cit1] Shahid M., Hussain A., Khan A. A., Alanazi A. M., Alaofi A. L., Alam M., Ramzan M. (2022). Antifungal Cationic Nanoemulsion Ferrying Miconazole Nitrate with Synergism to Control Fungal Infections: *In Vitro*, *Ex Vivo*, and *In Vivo* Evaluations. ACS Omega.

[cit2] Alyahya E. M., Alwabsi K., Aljohani A. E., Albalawi R., El-Sherbiny M., Ahmed R., Mortagi Y., Qushawy M. (2023). Preparation and Optimization of Itraconazole Transferosomes-Loaded HPMC Hydrogel for Enhancing Its Antifungal Activity: 2^3 Full Factorial Design. Polymers.

[cit3] Fisher M. C., Alastruey-Izquierdo A., Berman J., Bicanic T., Bignell E. M., Bowyer P., Bromley M., Brüggemann R., Garber G., Cornely O. A., Gurr S. J., Harrison T. S., Kuijper E., Rhodes J., Sheppard D. C., Warris A., White P. L., Xu J., Zwaan B., Verweij P. E. (2022). Tackling the emerging threat of antifungal resistance to human health. Nat. Rev. Microbiol..

[cit4] Teng F., Deng P., Song Z., Zhou F., Feng R. (2017). Enhanced effect in combination of curcumin- and ketoconazole-loaded methoxy poly(ethylene glycol)-poly(ε-caprolactone) micelles. Biomed. Pharmacother..

[cit5] Kumar A. , Panwar D., Bhavana V., Thakor P., Singh P. K. and Mehra N. K., Lipid-Based Nanomaterials: A Brief Note on Composition, Development, and Drug Delivery Applications, in Nanomaterial-Based Drug Delivery Systems: Therapeutic and Theranostic Applications, ed. C. V. Pardeshi, Springer International Publishing, Cham, 2023, pp. 65–98

[cit6] Sadozai S., Khan S., Karim N., Becker D., Steinbrück N., Gier S., Baseer A., Breinig F., Kickelbick G., Schneider M. (2020). Ketoconazole loaded nanoparticles and its synergism against Candida albicans when combined with silver nanoparticles. J. Drug Delivery Sci. Technol..

[cit7] Dudhipala N., Ay A. A. (2020). Amelioration of ketoconazole in lipid nanoparticles for enhanced antifungal activity and bioavailability through oral administration for management of fungal infections. Chem. Phys. Lipids.

[cit8] Ramzan M., Gourion-Arsiquaud S., Hussain A., Gulati J. S., Zhang Q., Trehan S., Puri V., Michniak-Kohn B., Kaur I. P. (2022). In vitro release, ex vivo penetration, and *in vivo* dermatokinetics of ketoconazole-loaded solid lipid nanoparticles for topical delivery. Drug Delivery Transl. Res..

[cit9] Ahmad I., Farheen M., Kukreti A., Afzal O., Akhter M. H., Chitme H., Visht S., Altamimi A. S. A., Alossaimi M. A., Alsulami E. R., Jaremko M., Emwas A. H. (2023). Natural Oils Enhance the Topical Delivery of Ketoconazole by Nanoemulgel for Fungal Infections. ACS Omega.

[cit10] Tiwari N., Sivakumar A., Mukherjee A., Chandrasekaran N. (2018). Enhanced antifungal activity of Ketoconazole using rose oil based novel microemulsion formulation. J. Drug Delivery Sci. Technol..

[cit11] Didehdar M., Chegini Z., Shariati A. (2022). Eugenol: A novel therapeutic agent for the inhibition of Candida species infection. Front. Pharmacol.

[cit12] Schmidt E., Jirovetz L., Wlcek K., Buchbauer G., Gochev V., Girova T., Stoyanova A., Geissler M. (2013). Antifungal Activity of Eugenol and Various Eugenol-Containing Essential Oils against 38 Clinical Isolates of Candida albicans. J. Essent. Oil Bear. Plants.

[cit13] Putta C. L., Rahman S. N. R., Chakraborty P., Shunmugaperumal T. (2023). Development, systematic optimisation and biofilm disruption activity of eugenol-based nanosized emulsions stabilised with Tween 80. J. Microencapsulation.

[cit14] Elgendy H. A., Makky A. M. A., Elakkad Y. E., Ismail R. M., Younes N. F. (2023). Syringeable atorvastatin loaded eugenol enriched PEGylated cubosomes *in situ* gel for the intra-pocket treatment of periodontitis: statistical optimization and clinical assessment. Drug Delivery.

[cit15] Grijalvo S., Rodriguez-Abreu C. (2023). Polymer nanoparticles from low-energy nanoemulsions for biomedical applications. Beilstein J. Nanotechnol..

[cit16] Liu T., Gao Z., Zhong W., Fu F., Li G., Guo J., Shan Y. (2022). Preparation, Characterization, and Antioxidant Activity of Nanoemulsions Incorporating Lemon Essential Oil. Antioxidants.

[cit17] Wooster T. J., Golding M., Sanguansri P. (2008). Impact of oil type on nanoemulsion formation and Ostwald ripening stability. Langmuir.

[cit18] Wik J., Bansal K. K., Assmuth T., Rosling A., Rosenholm J. M. (2020). Facile methodology of nanoemulsion preparation using oily polymer for the delivery of poorly soluble drugs. Drug Delivery Transl. Res..

[cit19] Pyrhönen J., Bansal K., Bhadane R., Wilen C.-E., Salo-Ahen O., Rosenholm J. (2021). Molecular Dynamics Prediction Verified by Experimental Evaluation of the Solubility of Different Drugs in Poly(decalactone) for the Fabrication of Polymeric Nanoemulsions. Adv. NanoBiomed Res..

[cit20] Maru S., Verma J., Wilen C. E., Rosenholm J. M., Bansal K. K. (2023). Attenuation of celecoxib cardiac toxicity using Poly(δ-decalactone) based nanoemulsion *via* oral route. Eur. J. Pharm. Sci..

[cit21] Bansal K. K., Gupta J., Rosling A., Rosenholm J. M. (2018). Renewable poly(δ-decalactone) based block copolymer micelles as drug delivery vehicle: *in vitro* and *in vivo* evaluation. Saudi Pharm. J..

[cit22] Bansal K., Kakde D., Purdie L., Irvine D., Howdle S., Mantovani G., Alexander C. (2015). New Biomaterials from Renewable Resources - Amphiphilic Block Copolymers from δ-Decalactone. Polym. Chem..

[cit23] Bansal K. K., Özliseli E., Saraogi G. K., Rosenholm J. M. (2020). Assessment of Intracellular Delivery Potential of Novel Sustainable Poly(δ-decalactone)-Based Micelles. Pharmaceutics.

[cit24] Inoue K., Ogawa K., Okada J., Sugibayashi K. (2005). Enhancement of skin permeation of ketotifen by supersaturation generated by amorphous form of the drug. J. Controlled Release.

[cit25] Sala M., Diab R., Elaissari A., Fessi H. (2018). Lipid nanocarriers as skin drug delivery systems: Properties, mechanisms of skin interactions and medical applications. Int. J. Pharm..

[cit26] Negi P., Singh B., Sharma G., Beg S., Katare O. P. (2015). Biocompatible lidocaine and prilocaine loaded-nanoemulsion system for enhanced percutaneous absorption: QbD-based optimisation, dermatokinetics and *in vivo* evaluation. J. Microencapsulation.

[cit27] Lambert E., Janjic J. M. (2021). Quality by design approach identifies critical parameters driving oxygen delivery performance *in vitro* for perfluorocarbon based artificial oxygen carriers. Sci. Rep..

[cit28] Tan S. F., Masoumi H. R., Karjiban R. A., Stanslas J., Kirby B. P., Basri M., Basri H. B. (2016). Ultrasonic emulsification of parenteral valproic acid-loaded nanoemulsion with response surface methodology and evaluation of its stability. Ultrason. Sonochem..

[cit29] Shi Y., Li H., Li J., Zhi D., Zhang X., Liu H., Wang H., Li H. (2015). Development, optimization and evaluation of emodin loaded nanoemulsion prepared by ultrasonic emulsification. J. Drug Delivery Sci. Technol..

[cit30] Ahmed M. M., Anwer M. K., Fatima F., Alali A. S., Kalam M. A., Zafar A., Alshehri S., Ghoneim M. M. (2022). Development of Apremilast Nanoemulsion-Loaded Chitosan Gels: *In Vitro* Evaluations and Anti-Inflammatory and Wound Healing Studies on a Rat Model. Gels.

[cit31] Abbas S., Karangwa E., Bashari M., Hayat K., Hong X., Sharif H. R., Zhang X. (2015). Fabrication of polymeric nanocapsules from curcumin-loaded nanoemulsion templates by self-assembly. Ultrason. Sonochem..

[cit32] Teaima M. H., Eltabeeb M. A., El-Nabarawi M. A., Abdellatif M. M. (2022). Utilization of propranolol hydrochloride mucoadhesive invasomes as a locally acting contraceptive: *in vitro*, *ex vivo*, and *in vivo* evaluation. Drug Delivery.

[cit33] Rosso A., Lollo G., Chevalier Y., Troung N., Bordes C., Bourgeois S., Maniti O., Granjon T., Dugas P.-Y., Urbaniak S., Briançon S. (2020). Development and structural characterization of a novel nanoemulsion for oral drug delivery. Colloids Surf., A.

[cit34] Chilamakuri S. N., Kumar A., Nath A. G., Gupta A., Selvaraju S., Basrani S., Jadhav A., Gulbake A. (2023). Development and *In Vitro* Evaluation of Eugenol-Based Nanostructured Lipid Carriers for Effectual Topical Treatment Against C. albicans. J. Pharm. Sci..

[cit35] Kumar A., Valamla B., Thakor P., Chary P. S., Rajana N., Mehra N. K. (2022). Development and evaluation of nanocrystals loaded hydrogel for topical application. J. Drug Delivery Sci. Technol..

[cit36] Verma D., Thakur P. S., Padhi S., Khuroo T., Talegaonkar S., Iqbal Z. (2017). Design expert assisted nanoformulation design for co-delivery of topotecan and thymoquinone: Optimization, *in vitro* characterization and stability assessment. J. Mol. Liq..

[cit37] Pourmadadi M., Ahmadi M., Abdouss M., Yazdian F., Rashedi H., Navaei-Nigjeh M., Hesari Y. (2022). The synthesis and characterization of double nanoemulsion for targeted Co-Delivery of 5-fluorouracil and curcumin using pH-sensitive agarose/chitosan nanocarrier. J. Drug Delivery Sci. Technol..

[cit38] Amoozegar H., Ghaffari A., Keramati M., Ahmadi S., Dizaji S., Moayer F., Akbarzadeh I., Abazari M., razzaghi-abyaneh M., Bakhshandeh H. (2022). A novel formulation of simvastatin nanoemulsion gel for infected wound therapy: *In vitro* and *in vivo* assessment. J. Drug Delivery Sci. Technol..

[cit39] Du X., Hu M., Liu G., Qi B., Zhou S., Lu K., Xie F., Zhu X., Li Y. (2022). Development and evaluation of delivery systems for quercetin: A comparative study between coarse emulsion, nano-emulsion, high internal phase emulsion, and emulsion gel. J. Food Eng..

[cit40] Vijaya Rani K. R., Rajan S., Bhupathyraaj M., Priya R. K., Halligudi N., Al-Ghazali M. A., Sridhar S. B., Shareef J., Thomas S., Desai S. M., Pol P. D. (2022). The Effect of Polymers on Drug Release Kinetics in Nanoemulsion *In Situ* Gel Formulation. Polymers.

[cit41] Rapalli V. K., Sharma S., Roy A., Alexander A., Singhvi G. (2021). Solid lipid nanocarriers embedded hydrogel for topical delivery of apremilast: *In vitro*, *ex vivo*, dermatopharmacokinetic and anti-psoriatic evaluation. J. Drug Delivery Sci. Technol..

[cit42] Teixeira A. D. R., Quaresma A. V., Branquinho R. T., Santos S., Magalhães J. T., Silva F., Marques M. B. F., Moura S. A. L., Barboza A. P. M., Araújo M. G. F., Silva G. R. D. (2023). Miconazole-loaded nanoparticles coated with hyaluronic acid to treat vulvovaginal candidiasis. Eur. J. Pharm. Sci..

[cit43] Pereira R. L., Leites F. I., Paese K., Sponchiado R. M., Michalowski C. B., Guterres S. S., Schapoval E. E. (2016). Hydrogel containing adapalene- and dapsone-loaded lipid-core nanocapsules for cutaneous application: development, characterization, *in vitro* irritation and permeation studies. Drug Dev. Ind. Pharm..

[cit44] Sivaraman A., Banga A. K. (2015). Quality by design approaches for topical dermatological dosage forms. Res. Rep. Transdermal Drug Delivery.

[cit45] Nikaeen G., Yousefinejad S., Rahmdel S., Samari F., Mahdavinia S. (2020). Central Composite Design for Optimizing the Biosynthesis of Silver Nanoparticles using Plantago major Extract and Investigating Antibacterial, Antifungal and Antioxidant Activity. Sci. Rep..

[cit46] Yang Z., Chai Y., Zhou D., Yao X., Ji H. (2019). Mechanism for efficient separation of eugenol and eugenol acetate with β -cyclodextrin as a selective solvent. Supramol. Chem..

[cit47] Nawaz A., Latif M. S., Alnuwaiser M. A., Ullah S., Iqbal M., Alfatama M., Lim V. (2022). Synthesis and Characterization of Chitosan-Decorated Nanoemulsion Gel of 5-Fluorouracil for Topical Delivery. Gels.

[cit48] Ramasamy T., Umadevi S., Ruttala H., Shanmugam S. (2012). Development of solid lipid nanoparticles enriched hydrogels for topical delivery of anti-fungal agent. Macromol. Res..

[cit49] Makuch E., Nowak A., Günther A., Pełech R., Kucharski Ł., Duchnik W., Klimowicz A. (2020). Enhancement of the antioxidant and skin permeation properties of eugenol by the esterification of eugenol to new derivatives. AMB Express.

[cit50] Raz-Pasteur A., Ullmann Y., Berdicevsky I. (2011). The pathogenesis of Candida infections in a human skin model: scanning electron microscope observations. ISRN Dermatol..

[cit51] Roberts M. S., Cheruvu H. S., Mangion S. E., Alinaghi A., Benson H. A. E., Mohammed Y., Holmes A., van der Hoek J., Pastore M., Grice J. E. (2021). Topical drug delivery: History, percutaneous absorption, and product development. Adv. Drug Delivery Rev..

[cit52] Osonga F. J., Eshun G., Kalra S., Yazgan I., Sakhaee L., Ontman R., Jiang S., Sadik O. A. (2022). Influence of Particle Size and Shapes on the Antifungal Activities of Greener Nanostructured Copper against Penicillium italicum. ACS Agric. Sci. Technol..

[cit53] Kathwate G. H., Shinde R. B., Karuppayil S. M. (2015). Antiepileptic Drugs Inhibit Growth, Dimorphism, and Biofilm Mode of Growth in Human Pathogen Candida albicans. Assay Drug Dev. Technol..

[cit54] Sanchez D. A., Schairer D., Tuckman-Vernon C., Chouake J., Kutner A., Makdisi J., Friedman J. M., Nosanchuk J. D., Friedman A. J. (2014). Amphotericin B releasing nanoparticle topical treatment of Candida spp. in the setting of a burn wound. Nanomedicine.

[cit55] Panácek A., Kolár M., Vecerová R., Prucek R., Soukupová J., Krystof V., Hamal P., Zboril R., Kvítek L. (2009). Antifungal activity of silver nanoparticles against Candida spp. Biomaterials.

[cit56] Oliveira A. C. S., Oliveira P. M., Cunha-Filho M., Gratieri T., Gelfuso G. M. (2020). Latanoprost Loaded in Polymeric Nanocapsules for Effective Topical Treatment of Alopecia. AAPS PharmSciTech.

[cit57] Balestrin L. A., Kreutz T., Fachel F. N. S., Bidone J., Gelsleichter N. E., Koester L. S., Bassani V. L., Braganhol E., Dora C. L., Teixeira H. F. (2021). Achyrocline satureioides (Lam.) DC (Asteraceae) Extract-Loaded Nanoemulsions as a Promising Topical Wound Healing Delivery System: *In Vitro* Assessments in Human Keratinocytes (HaCaT) and HET-CAM Irritant Potential. Pharmaceutics.

[cit58] Ruscinc N., Massarico Serafim R. A., Almeida C., Rosado C., Baby A. R. (2024). Challenging the safety and efficacy of topically applied chlorogenic acid, apigenin, kaempferol, and naringenin by HET-CAM, HPLC-TBARS-EVSC, and laser Doppler flowmetry. Front. Chem..

[cit59] da Costa B., Pippi B., Berlitz S. J., Carvalho A. R., Teixeira M. L., Külkamp-Guerreiro I. C., Andrade S. F., Fuentefria A. M. (2021). Evaluation of activity and toxicity of combining clioquinol with ciclopirox and terbinafine in alternative models of dermatophytosis. Mycoses.

