# Supplementary material for: A poly-δ-decalactone (PDL) based nanoemulgel for topical delivery of ketoconazole and eugenol against Candida albicans
Source: Nanoscale Adv. 2024 Aug 2;6(21):5322–36. doi: 10.1039/d4na00176a (PMC11376195; doi:10.1039/d4na00176a)
Supplement: NA-006-D4NA00176A-s001 [file NA-006-D4NA00176A-s001.pdf]

Supplementary Information (SI) for Nanoscale Advances.
This journal is © The Royal Society of Chemistry 2024

# Supporting Information

## 1. Methods

### 1.1. Synthesis and characterization of polymers

The homopolymer PDL and copolymer mPEG-b-PDL were synthesized using benzyl alcohol and $mPEG_{5k}$ as initiator according to the reported procedure. The synthesis and purification of polymers were confirmed by $^1$HNMR values [1].

### 1.2. *Ex vivo* permeation study

Fresh pig ear skin was collected from a local slaughterhouse. The skin was thoroughly washed with a buffer solution, the hair on it was removed with a surgical razor, the skin was separated from the underlying cartilage, and subcutaneous fat was removed with a scalpel [2]. Franz diffusion cell with an effective diffusion area of 3.14 $cm^2$ was utilized to conduct the *ex vivo* permeation study. The previously prepared skin was clamped between the donor and receptor chambers of the diffusion cell. The receptor chamber was filled with freshly prepared 10% methanol-added phosphate buffer (pH 5.5). The receptor diffusion cell was maintained at 37°C and a stirring rate of 200 rpm. The coarse KTZ-EUG-Gel and KTZ-EUG-NE-Gel were applied to the skin. A 1 ml sample at 0.5, 1, 2, 4, 8, and 12h time intervals was collected and replenished with the same amount to maintain sink conditions. The sample was filtered and analyzed using the validated RP-HPLC method. The experiment was performed in triplicate (n = 3), and data were plotted for the cumulative amount of drug permeated against time [3].

The values obtained from coarse-KTZ-EUG-Gel and KTZ-EUG-NE-Gel were compared using the steady-state transdermal flux (Jss) and permeability coefficient (Kp), which were calculated using **Eq. 1**. When plotting the cumulative amount of drug permeated ($cm^{-2}$) against time, Jss was found from the linear slope of the line. Using a flux to the drug concentration at the donor compartment (Co), Kp was calculated as follows:

$$Jss = \frac{dQ}{dt.A} \quad \textbf{(1)}$$

$$Kp = Jss/Co \quad \textbf{(2)}$$

### 1.3. Skin retention study

When the *ex vivo* permeation study was completed, the skin was taken out of the Franz diffusion cell. The non-permeated amount of gel was removed with a cotton swab and rinsed

with phosphate buffer saline (PBS) of pH 7.4. The tape-stripping method was utilized to investigate the drug retention at different superficial layers of the skin. The stratum corneum was removed using twelve tape strips. The dermis and epidermis were removed in the same manner and cut into pieces. The drug was extracted from the stripes and skin using methanol. The solution containing drugs was centrifuged, and the supernatant solution was filtered and analyzed using the developed RP-HPLC method [4].

In addition, the permeation or retention of KTZ or EUG was conducted after the preparation of fluorescent-loaded NE-Gel. The FITC was taken as a contrasting agent owing to lipophilic properties, mimicking KTZ and EUG. Thus, the permeation of FITC can be correlated to the *ex vivo* permeation of KTZ and EUG. In the previous *ex vivo* quantitative permeation study, the higher drug concentration was permeated after 12h. Thus, the fluorescent images of the skin were taken after 12h to investigate the FITC-NE-Gel permeation or retention. After the successful 12h permeation, the skin was removed, and processing was carried out as mentioned by Chwiej et al [5]. A 10µm section of skin was cut using a microtome (HistoCore Multicut, Leica). The section of skin was analyzed for permeation or retention of FITC using a fluorescence microscope (Invitrogen, ThermoFisher Scientific) under suitable magnification. The images were taken in brightfield and fluorescent view mode to determine the presence of FITC in the skin layers.

## 1.4. *In vitro* antifungal studies

### *1.4.1. Disk diffusion assay (DDA)*

DDA was conducted on yeast extract peptone dextrose agar plates. Briefly, the fungal strain ATCC 90028 was incubated on YPD medium and grown overnight at 30°C with agitation at 100 rpm. The cells were further pelleted, washed, and examined using a hemocytometer for cell count, followed by dilution with YPD agar medium. A 20µl of 1× $10^7$ *C. albicans* cells were transferred to YPD agar and transferred to 100-mm-diameter Petri dishes. The 10µlof each coarse KTZ-EUG dispersion, Blank NE, and KTZ-EUG-NE were spared on YPD plates and incubated at 30° C [4, 6, 7]. After 24h of incubation, the plates were analyzed for the zone of inhibition.

### *1.4.2. Effect of the formulations on planktonic growth*

The protocol employed to conduct planktonic growth strictly adhered to Clinical Laboratory Standards Institute (CLSI) guidelines. *C. albicans* cells (1×$10^3$ cells/mL) containing ten different concentrations of coarse KTZ-EUG dispersion, blank NE, and KTZ-EUG-NE (3,1.25,

0.625,0.312,0.156,0.078,0.039 and 0.019 µg/ml) were prepared in RPMI-1640. The well without drugs or formulations was treated as the control group. Concentrations containing 100 µl of *C. albicans* cells and 100 µl of coarse KTZ-EUG dispersion, blank NE, and KTZ-EUG-NE were added to a 96-well microtiter plate. The plate was incubated at 35°C for 48h and was analyzed by measuring the optical density at 620 nm using a microplate reader (Multiskan sky spectrophotometer). The concentration at which 50% growth was reduced was considered $MIC_{50}$ for planktonic growth [4, 7, 8]. The entire experiment was carried out in triplicates, and data were represented as mean ±SD.

### *1.4.3. Effect of formulations on yeast to hyphal morphogenesis (Y-H Morphogenesis)*

The effect of formulations on yeast to hyphal morphogenesis in *C. albicans* was studied using fetal bovine serum (FBS). The concentration range of coarse KTZ-EUG dispersion, blank NE, and KTZ-EUG-NE (3,1.25, 0.625,0.312,0.156,0.078,0.039 and 0.019 µg/ml) was prepared in 20% FBS, and cells without treatment were considered as controls. The plates containing $1\times10^6$ cells/ml cells were incubated at 37°C, with 100 rpm shaking for 90 min. The percentage of yeast and hyphal forms in each well was counted, and the concentration at which 50% yeast to hyphal formation was considered as $MIC_{50}$ for yeast to hyphal morphogenesis [4, 7, 8]. The experiment was performed in triplicate.

### *1.4.4. Effect of the formulation on Candida albicans biofilm formation*

To check the KTZ-EUG-NE activity against biofilm development, cells were allowed to adhere to the polystyrene surface of 96 well-treated plates for 90 min. The incubated wells were washed with sterile PBS and RPMI 1640 medium comprising coarse KTZ-EUG dispersion, blank NE, and KTZ-EUG-NE at different concentrations (3, 1.25, 0.625, 0.312, 0.156, 0.078, 0.039 and 0.019 µg/ml) was added to each well. The plate was incubated for 48h at 37°C, and biofilm growth was observed under an inverted microscope and quantified using the XTT metabolic assay [4, 7, 8].

## 2. Supplementary figures

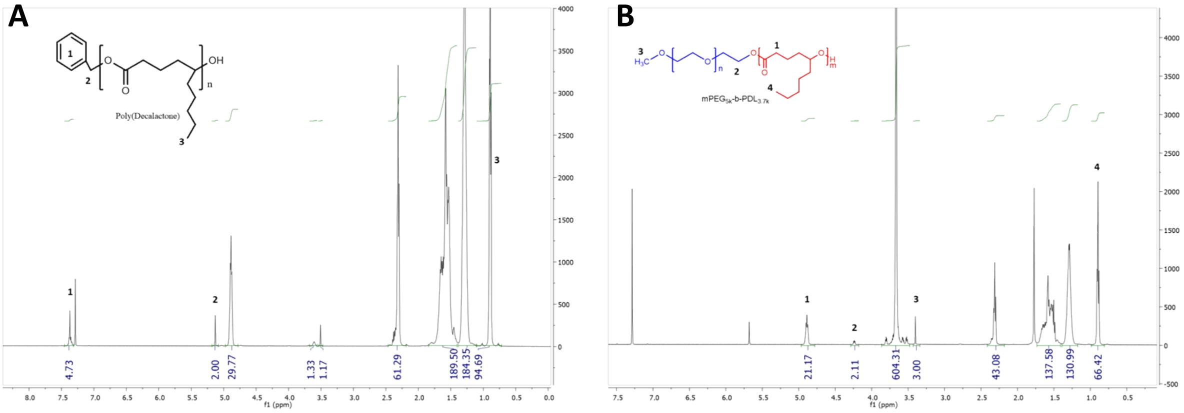


**Figure S1** $^{1}$HNMR spectra of **A.** PDL and **B.** mPEG-b-PDL in $CDCl_3$ acquired on Bruker's 500MHz.

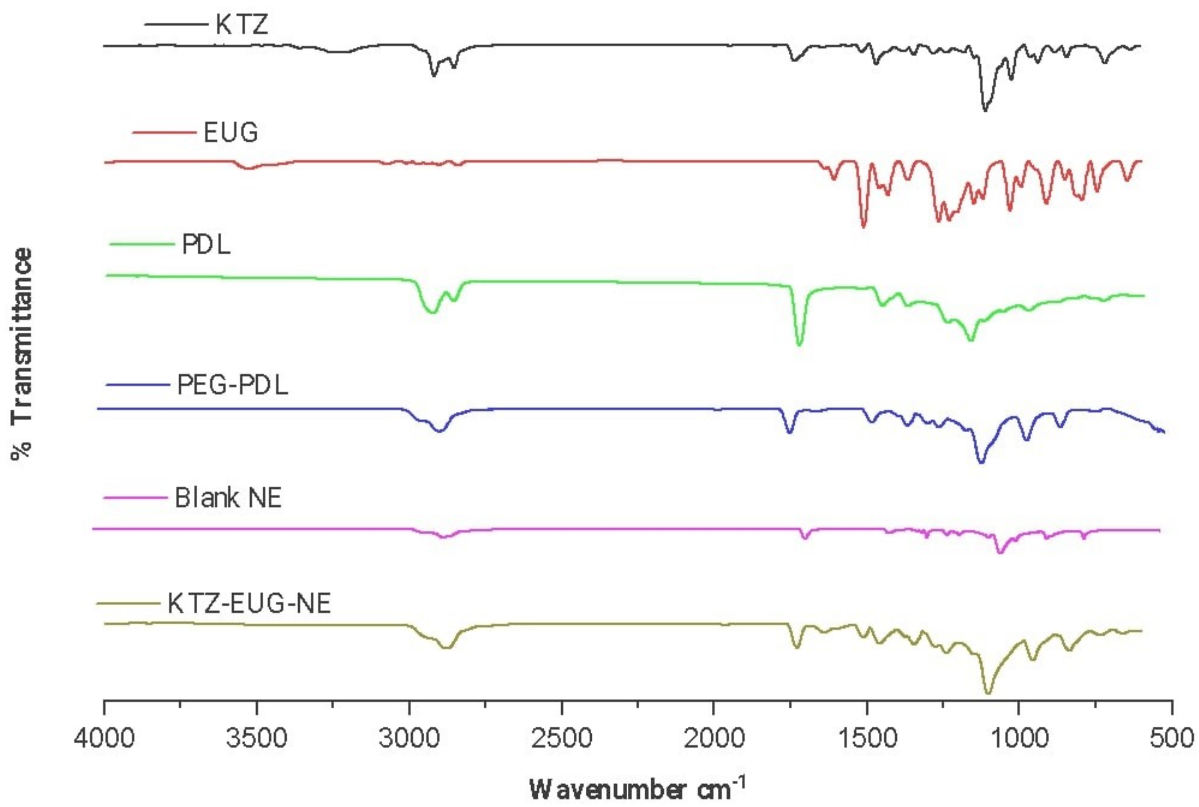


**Figure S2** ATR spectrum of KTZ, EUG, PDL, mPEG-b-PDL, Blank NE and KTZ-EUG-NE.

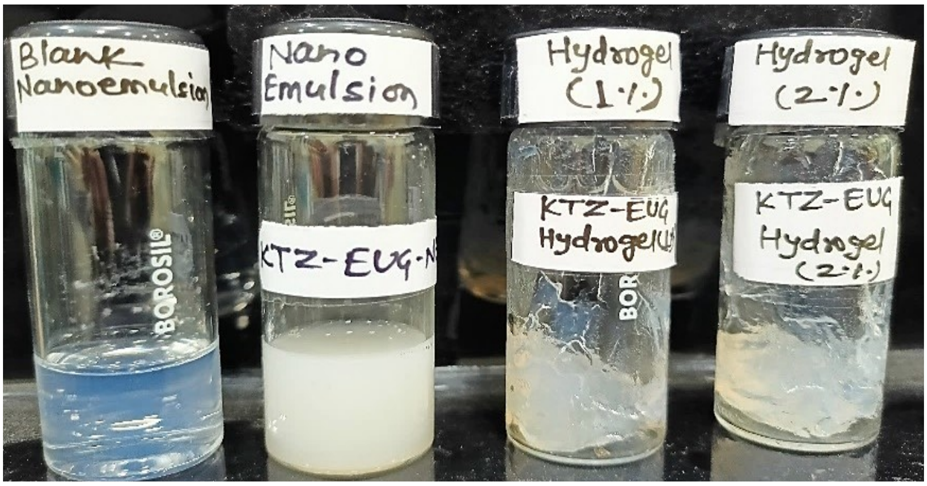


**Figure S3** Visual examination of blank NE, KTZ-EUG-NE, 1% KTZ-EUG-NE-Gel and 2% KTZ-EUG-NE-gel.

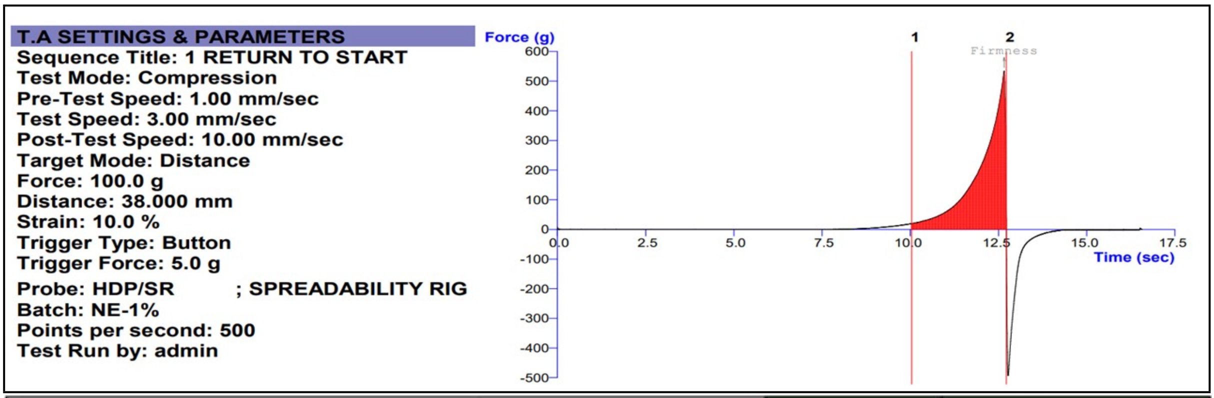


| Test ID | Batch | Firmness | Work of shear |
|---|---|---|---|
| | | g | g.sec |
| | | Firmness | a (Traditional) F-T |
| | | | |
| Start Batch NE-1% | NE-1% | | |
| NE-1%5 | NE-1% | 534.675 | 399.379 |
| End Batch NE-1% | NE-1% | | |

**Figure S4** Spreadability of the 1% KTZ-EUG-NE-Gel tested through texture analyzer (TA-XT plus).

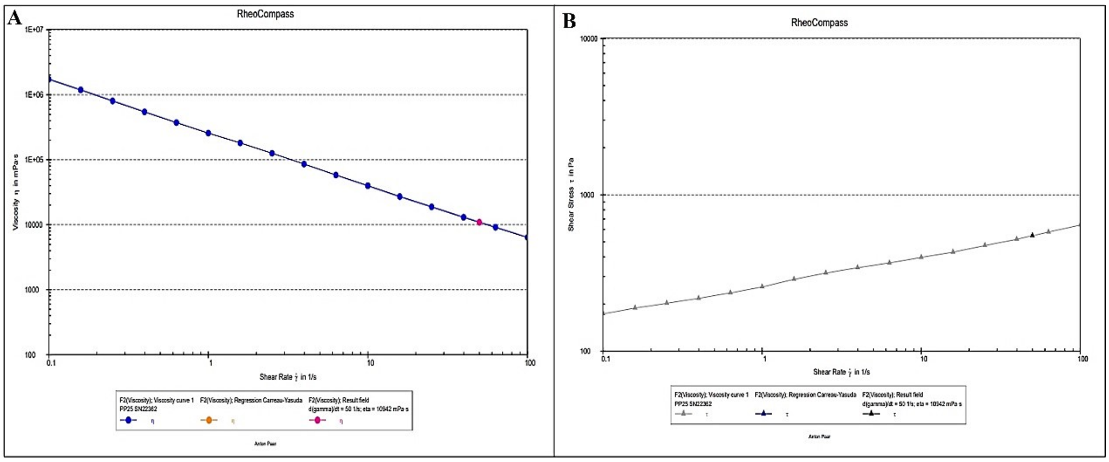


**Figure S5** Rheological behavior of optimized 1% w/w KTZ-EU-based NE hydrogel illustrating the correlation of shear rate versus **A.** viscosity **B.** Shear stress.

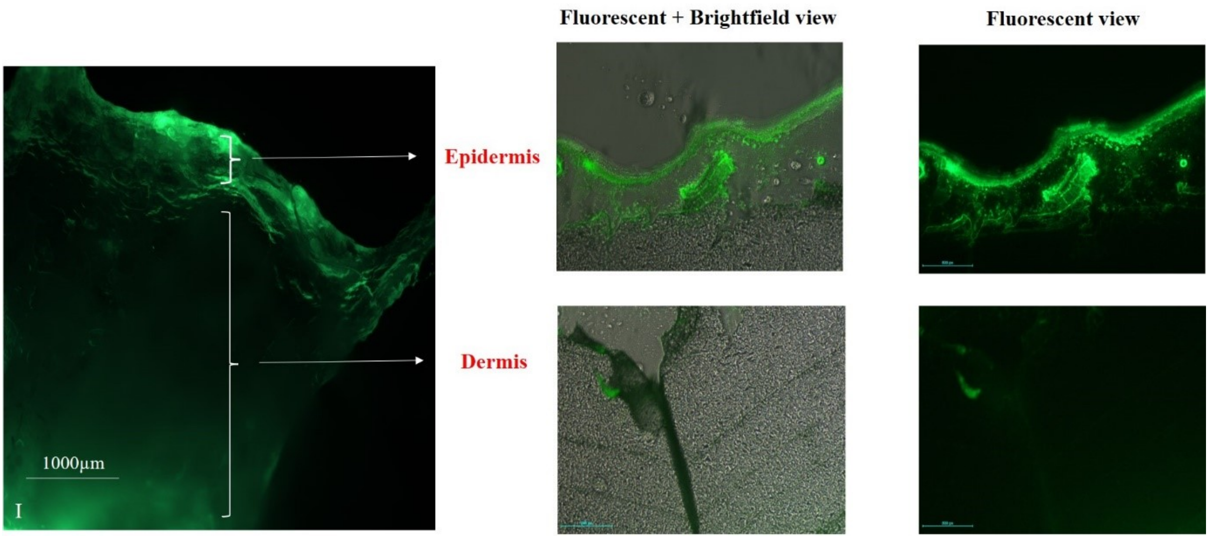


**Figure S6** *Ex vivo* permeation study after 12h illustrating I. The retention of FITC-NE-Gel in different layers of the skin, i.e., epidermis and dermis. The brightfield and fluorescent views represent the merged microscopic, focused section, while the green-fluorescent view represents the FITC-NE-Gel.

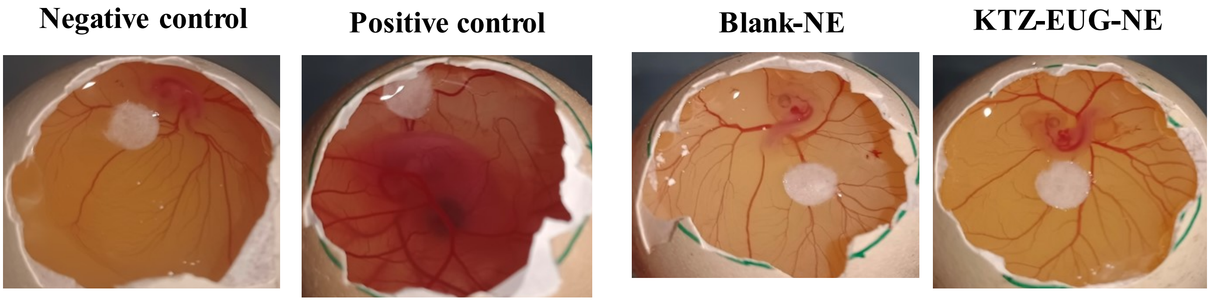


**Figure S7** Illustrates the effect of negative control (0.9% NaCl), positive control (300 µL of 0.1M NaOH), Blank NE, and KTZ-EUG-NE after treatment.

## 3. Supplementary tables

**Table S1. QTPP parameters concerning KTZ-EUG-NE**

| **Quality target product profile (QTPP)** | | |
|---|---|---|
| **Target Product Profile** | **Target** | **Justification** |
| Dosage form | KTZ-EUG-NE | For stability, faster-sustained release effect, and enhanced permeation |
| Route of administration | Topical when incorporated into the gelling system | To provide a better-localized effect, reduced systemic absorption |
| Dose strength | 0.1% w/w KTZ<br>0.05% w/v | Dose within the minimum effective concentration range |
| Appearance | Transparent | Free from grittiness, odor, or color, patient compliance |
| Globule size | < 200 nm | The lower globule size, enhance permeation from the stratum corneum |
| Drug content | Maximum | To enhance the efficacy |
| Release | Sustained release of drugs | Better drug release from the NE within the skin |

**Table S2. ANOVA table for the estimated globule size with CCD**

| Source | Sum of Squares | df | Mean Square | *F*-value | *p*-value | |
|---|---|---|---|---|---|---|
| **Model** | 54077.64 | 9 | 6008.63 | 54.53 | <0.0001 | Significant |
| A-Surfactant | 538.93 | 1 | 538.93 | 4.89 | 0.0514 | |
| B-Amplitude | 3578.45 | 1 | 3578.45 | 32.47 | 0.0002 | |
| C-Drug | 9742.27 | 1 | 9742.27 | 88.41 | <0.0001 | |
| AB | 1705.28 | 1 | 1705.28 | 15.47 | 0.0028 | |
| AC | 1123.38 | 1 | 1123.38 | 10.19 | 0.0096 | |
| BC | 6105.13 | 1 | 6105.13 | 55.40 | <0.0001 | |
| A2 | 11030.74 | 1 | 11030.74 | 100.10 | <0.0001 | |
| B2 | 18304.24 | 1 | 18304.24 | 166.10 | <0.0001 | |
| C2 | 7785.73 | 1 | 7785.73 | 70.65 | <0.0001 | |
| **Residual** | 1101.99 | 10 | 110.20 | | | |
| Lack of Fit | 904.72 | 5 | 180.94 | 4.59 | 0.0600 | Not significant |
| Pure Error | 197.28 | 5 | 39.46 | | | |
| **Cor Total** | 55179.63 | 19 | | | | |

**Table S3. ANOVA table for the estimate PDI with CCD**

| **Source** | **Sum of Squares** | **df** | **Mean Square** | ***F*-value** | ***p*-value** | |
|---|---|---|---|---|---|---|
| Model | 0.2182 | 9 | 0.0242 | 34.89 | < 0.0001 | significant |
| A-Surfactant | 0.0002 | 1 | 0.0002 | 0.2531 | 0.6258 | |
| B-Amplitude | 0.0156 | 1 | 0.0156 | 22.41 | 0.0008 | |
| C-Drug | 0.0808 | 1 | 0.0808 | 116.32 | < 0.0001 | |
| AB | 0.0003 | 1 | 0.0003 | 0.4680 | 0.5094 | |
| AC | 0.0096 | 1 | 0.0096 | 13.81 | 0.0040 | |
| BC | 0.0028 | 1 | 0.0028 | 3.99 | 0.0735 | |
| $A^2$ | 0.0291 | 1 | 0.0291 | 41.92 | < 0.0001 | |
| $B^2$ | 0.0122 | 1 | 0.0122 | 17.50 | 0.0019 | |
| $C^2$ | 0.0838 | 1 | 0.0838 | 120.59 | < 0.0001 | |
| **Residual** | 0.0069 | 10 | 0.0007 | | | |
| Lack of Fit | 0.0053 | 5 | 0.0011 | 3.34 | 0.1058 | not significant |
| Pure Error | 0.0016 | 5 | 0.0003 | | | |
| **Cor Total** | 0.2251 | 19 | | | | |

**Table S4. Different statistical parameters are given in the table regarding zeta potential**

| Source | Sum of Squares | df | Mean Square | *F*-value | *p*-value | |
|---|---|---|---|---|---|---|
| **Model** | 142.14 | 9 | 15.79 | 10.91 | 0.0004 | significant |
| A-Surfactant | 78.12 | 1 | 78.12 | 53.95 | < 0.0001 | |
| B-Amplitude | 8.34 | 1 | 8.34 | 5.76 | 0.0373 | |
| C-Drug | 12.37 | 1 | 12.37 | 8.54 | 0.0152 | |
| AB | 0.8871 | 1 | 0.8871 | 0.6127 | 0.4519 | |
| AC | 1.35 | 1 | 1.35 | 0.9310 | 0.3574 | |
| BC | 3.21 | 1 | 3.21 | 2.22 | 0.1675 | |
| $A^2$ | 5.88 | 1 | 5.88 | 4.06 | 0.0716 | |
| $B^2$ | 23.39 | 1 | 23.39 | 16.16 | 0.0024 | |
| $C^2$ | 4.84 | 1 | 4.84 | 3.34 | 0.0976 | |
| **Residual** | 14.48 | 10 | 1.45 | | | |
| Lack of Fit | 12.07 | 5 | 2.41 | 5.02 | 0.0506 | not significant |
| Pure Error | 2.40 | 5 | 0.4809 | | | |
| **Cor Total** | 156.62 | 19 | | | | |

**Table S5. Different statistical parameters are given in the table regarding drug content**

| Source | Sum of Squares | df | Mean Square | *F*-value | *p*-value | |
|---|---|---|---|---|---|---|
| **Model** | 1595.35 | 9 | 177.26 | 66.34 | < 0.0001 | significant |
| A-Surfactant (X1) | 86.49 | 1 | 86.49 | 32.37 | 0.0002 | |
| B-Amplitude (X2) | 80.86 | 1 | 80.86 | 30.26 | 0.0003 | |
| C-Drug (X3) | 1229.23 | 1 | 1229.23 | 460.03 | < 0.0001 | |
| AB | 5.22 | 1 | 5.22 | 1.95 | 0.1926 | |
| AC | 0.6962 | 1 | 0.6962 | 0.2605 | 0.6208 | |
| BC | 5.22 | 1 | 5.22 | 1.95 | 0.1926 | |
| $A^2$ | 18.18 | 1 | 18.18 | 6.80 | 0.0261 | |
| $B^2$ | 18.01 | 1 | 18.01 | 6.74 | 0.0267 | |
| $C^2$ | 171.48 | 1 | 171.48 | 64.18 | < 0.0001 | |
| **Residual** | 26.72 | 10 | 2.67 | | | |
| Lack of Fit | 18.62 | 5 | 3.72 | 2.30 | 0.1912 | not significant |
| Pure Error | 8.10 | 5 | 1.62 | | | |
| **Cor Total** | 1622.07 | 19 | | | | |

**Table S6. In-vitro drug release kinetic models**

| Drug | Zero-order | | | First-order | | | Higuchi | | | Korsmeyer-Peppas | | |
|---|---|---|---|---|---|---|---|---|---|---|---|---|
| | $R^2$ | AIC | $K_0$ | $R^2$ | AIC | $K_1$ | $R^2$ | AIC | $K_H$ | $R^2$ | AIC | $K_{kp}$ and (n) |
| **KTZ** | 0.70 | 61.30 | 3.896 | 0.95 | 46.55 | 0.083 | 0.93 | 49.50 | 16.152 | 0.965 | 45.12 | 14.59 (0.54) |
| **EUG** | -10.4 | 64.71 | 9.272 | 0.42 | 59.52 | 0.065 | 0.83 | 49.58 | 14.43 | 0.964 | 39.15 | 21.41 (0.468) |

## References


[1] K. Bansal, D. Kakde, L. Purdie, D. Irvine, S. Howdle, G. Mantovani, C. Alexander, New Biomaterials from Renewable Resources - Amphiphilic Block Copolymers from δ-Decalactone, Polym Chem, 6 (2015).

[2] B. Alhasso, M.U. Ghori, B.R. Conway, Development of Nanoemulsions for Topical Application of Mupirocin, Pharmaceutics, 15 (2023).

[3] S. Chaturvedi, A. Garg, Development and optimization of nanoemulsion containing exemestane using box-behnken design, J Drug Deliv Sci Technol, 80 (2023) 104151.

[4] E.H. Endo, R.Y. Makimori, M.V.P. Companhoni, T. Ueda-Nakamura, C.V. Nakamura, B.P. Dias Filho, Ketoconazole-loaded poly-(lactic acid) nanoparticles: Characterization and improvement of antifungal efficacy in vitro against Candida and dermatophytes, J Mycol Med, 30 (2020) 101003.

[5] J. Chwiej, M. Szczerbowska-Boruchowska, M. Lankosz, S. Wojcik, G. Falkenberg, Z. Stegowski, Z. Setkowicz, Preparation of tissue samples for X-ray fluorescence microscopy, Spectrochim Acta Part B At Spectrosc, 60 (2005) 1531-1537.

[6] B. Tian, Q. Yan, J. Wang, C. Ding, S. Sai, Enhanced antifungal activity of voriconazole-loaded nanostructured lipid carriers against Candida albicans with a dimorphic switching model, Int J Nanomedicine, 12 (2017) 7131-7141.

[7] S.N. Chilamakuri, A. Kumar, A.G. Nath, A. Gupta, S. Selvaraju, S. Basrani, A. Jadhav, A. Gulbake, Development and In-Vitro Evaluation of Eugenol-Based Nanostructured Lipid Carriers for Effectual Topical Treatment Against C. albicans, J Pharm Sci, 113 (2023) 772-784.

[8] K.M. Hamad, N.N. Mahmoud, S. Al-Dabash, L.A. Al-Samad, M. Abdallah, A.G. Al-Bakri, Fluconazole conjugated-gold nanorods as an antifungal nanomedicine with low cytotoxicity against human dermal fibroblasts, RSC Adv, 10 (2020) 25889-25897.
